# Interaction between glycolysis‒cholesterol synthesis axis and tumor microenvironment reveal that gamma-glutamyl hydrolase suppresses glycolysis in colon cancer

**DOI:** 10.3389/fimmu.2022.979521

**Published:** 2022-12-07

**Authors:** Yan-Jie Chen, Xi Guo, Meng-Ling Liu, Yi-Yi Yu, Yue-Hong Cui, Xi-Zhong Shen, Tian-Shu Liu, Li Liang

**Affiliations:** ^1^ Department of Gastroenterology, Zhongshan Hospital Fudan University, Shanghai, China; ^2^ Department of Medical Oncology, Zhongshan Hospital Fudan University, Shanghai, China; ^3^ Cancer Center, Zhongshan Hospital Fudan University, Shanghai, China; ^4^ Center of Evidence-based Medicine, Zhongshan Hospital Fudan University, Shanghai, China

**Keywords:** glycolysis‒cholesterol synthesis axis, metabolic subtypes, prognosis, tumor microenvironment, gamma-glutamyl hydrolase (GGH), colon cancer (CC)

## Abstract

**Background:**

Metabolic reprogramming is a feature of cancer. However, colon cancer subtypes based on the glycolysis‒cholesterol synthesis axis have not been identified, and little is known about connections between metabolic features and the tumor microenvironment.

**Methods:**

Data for 430 colon cancer cases were extracted from The Cancer Genome Atlas, including transcriptome data, clinical information, and survival outcomes. Glycolysis and cholesterol synthesis-related gene sets were obtained from the Molecular Signatures Database for a gene set variation analysis. The relationship between the genomic landscape and immune landscape were investigated among four metabolic subtypes. Hub genes were determined. The clinical significance of candidate hub gene was evaluated in 264 clinical samples and potential functions were validated *in vitro* and *in vivo*.

**Results:**

Colon cancer cases were clustered into four metabolic subtypes: quiescent, glycolytic, cholesterogenic, and mixed. The metabolic subtypes differed with respect to the immune score, stromal score, and estimate score using the ESTIMATE algorithm, cancer-immunity cycle, immunomodulator signatures, and signatures of immunotherapy responses. Patients in the cholesterogenic group had better survival outcomes than those for other subtypes, especially glycolytic. The glycolytic subtype was related to unfavorable clinical characteristics, including high mutation rates in TTN, APC, and TP53, high mutation burden, vascular invasion, right colon cancer, and low-frequency microsatellite instability. *GGH*, *CACNG4*, *MME*, *SLC30A2*, *CKMT2*, *SYN3*, and *SLC22A31* were identified as differentially expressed both in glycolytic-cholesterogenic subgroups as well as between colon cancers and healthy samples, and were involved in glycolysis‒cholesterol synthesis. GGH was upregulated in colon cancer; its high expression was correlated with CD4^+^ T cell infiltration and longer overall survival and it was identified as a favorable independent prognostic factor. The overexpression of GGH in colon cancer-derived cell lines (SW48 and SW480) inhibited PKM, GLUT1, and LDHA expression and decreased the extracellular lactate content and intracellular ATP level. The opposite effects were obtained by GGH silencing. The phenotype associated with GGH was also validated in a xenograft nude mouse model.

**Conclusions:**

Our results provide insight into the connection between metabolism and the tumor microenvironment in colon cancer and provides preliminary evidence for the role of GGH, providing a basis for subsequent studies.

## Background

According to GLOBOCAN, there were 1.14 million new cases and 0.56 million deaths from colon cancer worldwide in 2020, ranking fifth and sixth among common malignant tumors in incidence and mortality, respectively (1). The disease-free survival rate is approximately 75% after radical surgery followed by chemotherapy for stage III colon cancer (2), while the median progression-free survival and median overall survival are approximately 10 months and 25–28 months, respectively, for patients with stage IV disease receiving systemic treatment (3). Biomarker signatures and clinical characteristics, such as RAS mutations, microsatellite status, tumor mutational burden (TMB), consensus molecular subtype (CMS), location, and physical activity, contribute to the stratification of patients expected to benefit from anticancer therapy (4–7)

The reprogramming of energy metabolism is a distinct hallmark of cancer (8), and proteins or enzymes involved in glycolysis and cholesterol synthesis play an important role in tumorigenesis (9, 10) **(** **Figure S1A** **)**. Hence, metabolite-driven genes may be used as predictive biomarkers for clinical applications. For example, Karasinska stratified pancreatic cancer into four subgroups (quiescent, glycolytic, cholesterogenic, and mixed) based on genomic and transcriptomic alterations and determined their prognostic value (11). Wang reported that the glycolysis–cholesterol synthesis axis is associated with survival outcomes in pan-gynecological cancer (12). Metabolic profiles and subtypes have also been surveyed in triple-negative breast cancer, cutaneous melanoma, and gastric cancer, and the glycolytic subgroup always exhibits an inferior survival rate compared to that of the cholesterogenic subgroup (13–15).

Cancer cells reprogram their microenvironment to survive and thrive in a way that influences their metabolic composition in the extracellular context. High levels of glucose and glutamine intake followed by lactate and CO_2_ output result in extracellular lactate accumulation and acidification, thereby weakening dendritic or T cell activation and monocyte migration (16–18), inducing macrophage M2-like conversion (19), promoting VEGF secretion from endothelial cells (20), and generating fibroblast-derived hyaluronic acid to facilitate tumor invasion (21). Reciprocally, the microenvironment influences tumor metabolism, tumor growth, and treatment responses. Restoring the glucose supply in tumor-infiltrating lymphocytes can increase the efficacy of checkpoint inhibitors in cancer immunotherapy. Chang et al. reported that glucose consumption by sarcoma cells metabolically represses T cell function and glycolysis, whereas checkpoint inhibitors restore glucose metabolism and promote glycolysis along with IFN-γ production in T cells (22). Ho et al. found a decrease in glucose uptake and the upregulation of immunosuppressive molecules (IFN-γ and CD40L) in CD4^+^ T cells in melanoma and identified the glycolytic enzyme hexokinase 2 (HK-2) in tumors and glycolytic metabolite phosphoenolpyruvate carboxy kinase 1 (PET-1) in T cells as key regulators in the interaction (23). Alterations in metabolite-driven gene regulation and metabolic competition between tumors and their microenvironment are emerging traits related to malignant tumor growth (24).

Glutamine and glucose are critical substrates in cancer cell metabolism. Glutamine supplies carbon and nitrogen for the synthesis of nucleotides, glutathione, and other metabolic substrates required for cancer cell development. A novel glutamine antagonist, JHU083, reduces tumor growth by inhibiting glutamine uptake for anabolism in cancer cells and enhancing the cell killing ability of CD8^+^ T cells in the microenvironment, providing a new therapeutic strategy based on glutamine metabolism (25). Venneti et al. evaluated the application of glutamine-based positron emission tomography imaging for the diagnosis of malignant tumors, revealing promising results (26).

In this study, we clustered colon cancer cases based on glycolysis- and cholesterol synthesis-related genes. We investigated genetic variation and transcriptional alterations in the metabolic subtypes and analyzed their relationships with the tumor microenvironment as well as survival outcomes. We also identified key genes involved in glycolysis‒cholesterol synthesis and validated the gamma-glutamyl hydrolase (GGH) phenotype for future mechanistic studies. A flowchart of our study is shown in **Figure S1B**.

## Materials and methods

### Data source

The raw transcriptome data and clinical information for colon cancer were retrieved from the GDC Data Portal of The Cancer Genome Atlas Colon Adenocarcinoma (TCGA-COAD, https://portal.gdc.cancer.gov/). Fragments per kilobase of transcript per million reads sequenced were translated into reads per kilobase million. Data for survival outcomes, methylation status, and copy number variation (CNV) were obtained from UCSC XENA (https://xena.ucsc.edu/). Somatic point mutation data, as single-nucleotide variants (SNVs), were obtained from cBioPortal (http://www.cbioportal.org/). Transcriptomic data for colon cancer cell lines and single-cell RNA sequencing data were downloaded from the Caner Cell Line Encyclopedia database (https://sites.broadinstitute.org/ccle) and GSE178341 of the Gene Expression Omnibus (GEO, https://www.ncbi.nlm.nih.gov/geo/). Other clinical cases were derived from the GSE39582 dataset.

### Genes involved in the glycolysis‒cholesterol synthesis axis

The gene sets REACTOME_GLYCOLYSIS and REACTOME_CHOLESTEROL_BIOSYNTHESIS were obtained from the Molecular Signatures Database (MsigDB) database (http://www.gsea-msigdb.org/gsea/index.jsp). A gene set variation analysis (GSVA) and differential expression analysis were executed using the GSVA package and limma package in R. For SNVs, mutect2 files were downloaded and mutation landscapes were visualized using the R MafTools package. Protein–protein interactions were studied using the STRING database and network clustering was performed using a Markov clustering algorithm. A principal component analysis (PCA) of subtypes was performed using the R PCAtools package and clinical survival outcomes were analyzed using the R survival package.

### Metabolism in the tumor immune microenvironment

The tumor immune microenvironment was evaluated by the immune score, stromal score, and ESTIMATE score using the ESTIMATE algorithm. A single-sample gene set enrichment analysis (ssGSEA) was employed to inspect the cancer-immunity cycle. Data for immunomodulators were derived from Charoentong et al. (27).

### Functional enrichment analysis and molecular typing

Gene Ontology (GO) and Kyoto Encyclopedia of Genes and Genomes (KEGG) pathway enrichment analyses as well as a gene set enrichment analysis (GSEA) were performed using the R clusterProfiler package. A pre-defined gene set (h.all.v7.symbols.gmt) was retrieved from the MSigDB (http://software.broadinstitute.org/gsea/msigdb). The thresholds for significant enrichment were a false discovery rate (FDR) of less than 0.25 and adjusted *p*-value of less than 0.05. Molecular subtypes for colon cancer were based on Kabbarah (6), Laird (28), and Shmulevich (29).

### Identification of hub genes involved in glucose and lipid metabolism

Thresholds for differentially expressed genes (DEGs) were an FDR-adjusted *p*-value of less than 0.05 and |Log_2_ Fold change (FC) | > 1, as evaluated using the R DEseq2 package. A weighted gene co-expression network analysis (WGCNA) was performed using the R WGCNA package.

### Patients and immunohistochemical analyses of tissue microarrays

Human colon cancer tissue microarrays were evaluated for 264 cases using paraffin-embedded human colon cancer samples from patients who received surgery or systemic chemotherapy at Zhongshan Hospital, Fudan University from January 2007 to March 2017. There were 259 and 154 cases of successful immunohistochemical staining in cancerous tissues and corresponding para-cancerous tissues, respectively. Clinical characteristics were recorded and are listed in **Table 1**. The follow-up period lasted until September 2020. Patients agreed to participate in the study and signed an informed consent document. The study was approved by the ethics committee of the Zhongshan Hospital. Tissue microarrays were prepared by the Department of Pathology at Zhongshan Hospital under standard operating conditions. Colon cancer and normal tissues were fixed using paraformaldehyde, embedded in paraffin, sliced into sections, and placed on glass slides. Then, the sections were subjected to deparaffinization, hydrophilization, and unmasking and were blocked with bovine serum albumin, stained with primary antibodies against GGH (bs-20360, 1:100, Bioss Biological Technology Co., Ltd., Beijing, China), CD4 (GB13064, 1:100, Servicebio Technology Co., Ltd., Wuhan, China), CD8 (GB13068, 1:100; Servicebio Technology Co., Ltd.), CD19 (GB11061, 1:500; Servicebio Technology Co., Ltd.), CD21 (GB13031, 1:300; Servicebio Technology Co., Ltd.), CD68 (GB13067, 1:100; Servicebio Technology Co., Ltd.), and MPO (GB11224, 1:500; Servicebio Technology Co., Ltd.). The samples were kept overnight at 4°C and subsequently incubated with a goat anti-rabbit secondary antibody (GB23303, 1:200; Servicebio Technology) for 30 min at 20°C. Stains were identified using a panoramic slice scanner (3DHISTECH, Budapest, Hungary), recorded, and imaged using CaseViewer 2.2 (3DHISTECH). The expression levels of GGH and other markers were evaluated by the H-score using Quant Center 2.1 (3DHISTECH). The H-score was calculated as follows: H-SCORE = ∑ (PI × I) = (percentage of cells with moderate intensity × 2) + percentage of cells with strong intensity × 3). “PI” indicates the proportion of the positive signal pixel area, whiles “I” indicates the color intensity.

**Table 1:** Basic data for 264 patients whose tissue samples were examined by immunohistochemical staining.

Characteristics	Number of cases	Percentage
**Gender**		
Male	147	55.7%
Female	117	44.3%
**Age**		
< 60	104	39.4%
≥ 60	160	60.6%
**Primary colon cancer site**		
Right	103	39.0%
Left	161	61.0%
**Radical surgery treatment**		
Yes	177	67.0%
No	87	33.0%
**T stage**		
T1	21	8.0%
T2	78	29.5%
T3	77	29.2%
T4	88	33.3%
**N stage**		
N0	68	25.8%
N1	92	34.8%
N2	104	
**M stage**		
M0	177	67.0%
M	87	33.0%
**KRAS mutation**		
Yes	135	51.1%
No	129	48.9%
**Survival status**		
Alive	173	65.5%
Death	80	30.3%
Censored	11	4.2%
**Successfully immunohistochemical staining**		
GGH in cancer	259	98.1%
GGH in para-cancer tissue	154	58.3%
**Total Number**	264	100.0%

### Cell culture conditions and lentivirus transduction

Cell lines, including NCM460, LOVO, CACO-2, SW48, and SW480, were purchased from the Cell Bank of the Chinese Academy of Sciences (Shanghai, China). Cells were cultured in Dulbecco’s modified Eagle medium (HyClone, Logan, UT, USA) containing 10% fetal bovine serum (Gibco, Paisley, UK) and were maintained in an incubator with 5% CO_2_ at 37°C. The cell lines were validated using short tandem repeat profiling (Genetic Testing Biotechnology Corporation, Suzhou, China) and routinely checked for *Mycoplasma* using the MycoAlert Mycoplasma Detection Kit (Lonza; LT07-218, Rockland, ME, USA). For GGH knockdown, three human siRNA sequences, including RNAi-1 (AAGAAGCCCATCATCGGAATATTAA), RNAi-2 (AAGATACTATATTGCTGCGTCCTAT), RNAi-3 (TACTATATTGCTGCGTCCTATCTAA) and a normal control, RNAi-nc (TTCTCCGAACGTGTCACGT), were synthesized by Genewiz Company (Shanghai, China) and cloned into the pLenti6.3 to generate pLenti6.3-GGH-RNAi. To overexpress GGH, the cDNAs of GGH (NM_003878) were synthesized and cloned into the pLenti6.3/V5-DEST vectors (Invitrogen, Carlsbad, CA, USA) to construct the overexpression plasmid. The integrity of the core plasmids was confirmed by DNA sequencing (Majorbio, Shanghai, China). The constructed plasmids and viral packaging plasmids were co-transfected into 293T cells to generate relevant lentiviruses, and supernatants were collected after 48 h of infection. SW48 and SW480 cells were inoculated in 6-well plates overnight before transduction, transfected with the lentivirus, and assigned to Control, OE-NC, OE-GGH, shNC, shGGH-1, and shGGH-2 groups at a multiplicity of infection of 5. Next, GGH mRNA and protein expression levels were determined by RT-PCR and western blotting. The transfected cells were continuously cultured and screened for 14 days for the insertion of target fragments and stable expression.

### Subcutaneous xenograft in nude mice

Male BALB/c nude mice (6 to 8 weeks of age, weighing approximately 20 g) were acquired from Suzhou Cavens Biogle Model Animal Research Co., Ltd. (Suzhou, China). Mice were housed in a specific pathogen-free facility and separated into four groups with five mice each. Xenograft experiments were performed with the approval of the Animal Experiments Ethics Committee of Zhongshan Hospital, Fudan University. The right backs of the mice were subcutaneously injected with 2 × 10^6^ SW480 cells transduced with OE-NC, OE-GGH, shNC, and shGGH. The length (L) and width (W) of the subcutaneous tumors were monitored and measured every 3 days using a Vernier caliper. The mice were sacrificed 4 weeks later and photographed, and the tumor tissues were dissected, weighed, and recorded. The tumor volume was calculated as follows: volume = (length × width^2^) × 0.5. The tumor lysates were analyzed to evaluate the expression levels of GGH, PKM, GLUD1, and LDHA by RT-qPCR and western blotting.

### RNA extraction, complementary DNA synthesis, and real-time quantitative polymerase chain reaction

Total RNA was extracted from cultured cell lines or xenograft models using TRIzol reagent (Invitrogen) and recombinant DNase I (Takara Bio Inc., Shiga, Japan), according to the manufacturers’ protocols. First-strand cDNA synthesis was achieved using the PrimeScript RT Master Mix Kit (Takara Bio Inc.). RT-qPCRs were performed on an ABI-7300 (ABI, Waltham, MA, USA) using the TB Green Premix Ex Taq Kit (Takara Bio Inc.). The cycling conditions were set as follows: pre-denaturation at 95°C for 10 min, followed by 40 cycles of denaturation at 95°C for 3 s, annealing at 60°C for 40 s, and elongation at 60°C for 50 s. The experiments were repeated three times in triplicate. Primers used for RT-qPCR are listed in **Supplementary Table S8**. All quantifications were normalized to β-actin as the internal reference, and the relative threshold cycle (Ct) was calculated as 2*^−ΔΔCt^*.

### Protein extraction and western blotting

Western blotting was performed on cultured cells or tissue samples after the indicated treatments. Cell lysates were collected using a sodium dodecyl sulfate lysis buffer (Beyotime Biotechnology, Shanghai, China). Equal amounts of total protein (approximately 15 μg for cell samples and 60 μg for tissue samples) were separated by 10% sodium dodecyl sulfate polyacrylamide gel electrophoresis and transferred onto nitrocellulose membranes (Millipore, Billerica, MA, USA). Membranes were blocked using 5% nonfat powdered milk (Sangon, Shanghai, China) in TBS with Tween-20 (TBST) at about 20°C for 1.5 h, incubated with primary antibodies overnight at 4°C, washed three times with TBST, and then incubated with secondary antibodies for 1 h. The signal intensity of protein bands was visualized using an Enhanced Chemiluminescence Detection Kit (Tanon, Shanghai, China). A semi-quantitative evaluation of protein density was performed using ImageJ (Version 1.5.3). The following antibodies were used at a 1:1000 dilution: anti-GGH (Cat. #138495; Abcam, Cambridge, UK), anti-PKM (Cat. #150377; Abcam), anti-GLUT1 (Cat. #115730; Abcam), anti-LDHA (Cat. #52488; Abcam), anti-β-actin (Cat. #4970, Abcam), and anti-GAPDH (Cat. #2118; Cell Signaling, Danvers, MA, USA).

### Cellular lactate and ATP content detection

The concentrations of lactic acid and ATP were detected using the Lactate Colorimetric Assay Kit (E-BC-F002-M; Elabscience Biotechnology Co., Ltd., Wuhan, China) and ATP Colorimetric Assay Kit (E-BC-K157-M; Elabscience Biotechnology Co., Ltd.). According to the manufacturer’s protocol, cells were seeded on 96-well plates and the optical density was measured at 530 nm and 636 nm. The lactate production and ATP levels in cells were calculated using as follows: Lactate (mmol/L) = [(ODtest - ODzero)/(ODstandard - ODzero)] × standard sample concentration (3 mmol/L) × sample dilution multiplier; ATP (mmol/L) = [(ODtest - ODcontrol)/(ODstandard - ODzero)] × standard sample concentration (1 mmol/L)/(1/volume of reagent) × dilution factor of sample before added to the system.

### Statistical methods

Data are presented as the mean ± standard deviation (SD). The Wilcoxon rank-sum test or Student’s *t*-test was used to compare two groups of data. Clinical data were analyzed using the chi-squared test. Correlations were evaluated by Spearman’s coefficient coefficients. A survival analysis was executed using Kaplan–Meier and log-rank tests. Cox regression analysis was used for univariate and multivariate analyses. Values of *p* < 0.05 were considered significant.

## Results

### Genomic variation, transcriptomic alterations, protein interactions for genes related to glycolysis–cholesterol synthesis, and patient clustering

**Figure 1:**
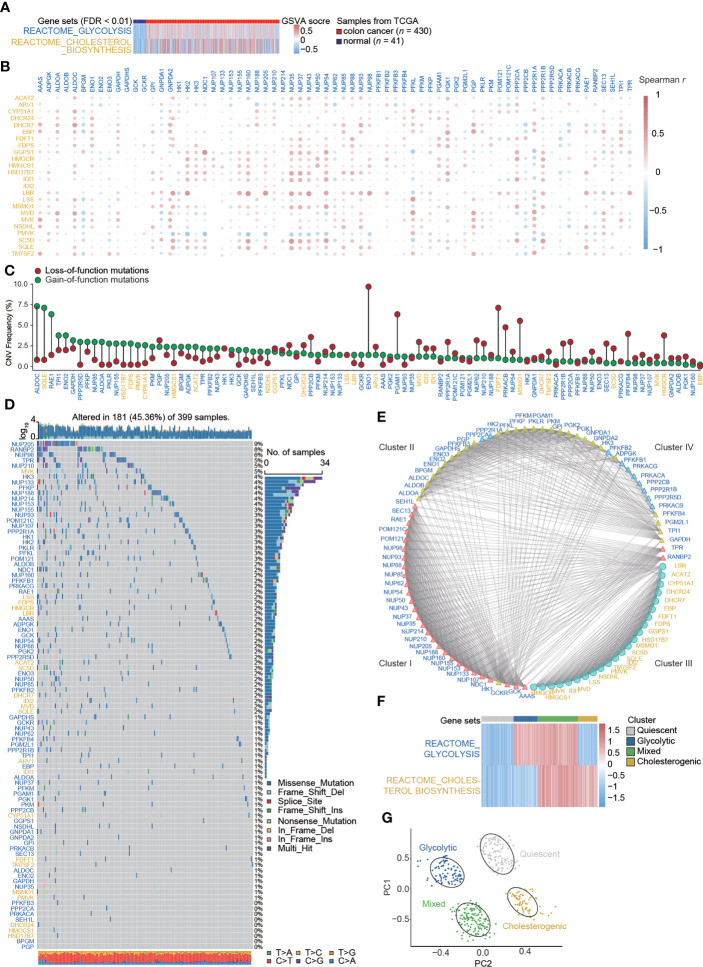
Signatures of the glycolysis–cholesterol synthesis axis and metabolic subtypes in colon cancer. **(A)** Enrichment for REACTOME_GLYCOLYSIS and REACTOME_CHOLESTEROL_ BIOSYNTHESIS differed between colon cancer and adjacent normal tissues. **(B)** Co-expression relations between signatures mediating glycolysis (labeled in blue) and cholesterol synthesis (labeled in yellow). **(C)** Lollipop plot showing copy number variation (CNV) represented by gains and losses. **(D)** Water plot showing single nucleotide variants (SNVs). **(E)** Interaction of signatures visualized by a protein–protein interaction network and stratified into four clusters. Glycolysis-related genes were distributed into three clusters (Cluster I, II, and IV), while cholesterol–synthesis-related genes were distributed into one cluster (Cluster III). **(F)** A hierarchical cluster analysis revealed four metabolic subtypes in colon cancer. **(G)** A principal component analysis suggested that colon cancer cases could be distinguished by metabolic subtypes.

REACTOME_GLYCOLYSIS and REACTOME_CHOLESTEROL_BIOSYNTHESIS were enriched in colon cancer, and GSVA scores were higher in colon cancer tissues than in paired normal tissues **(** **Figures 1A**, **S2** **)**. Sixty-nine glycolysis-related genes (labeled in blue) and twenty-four cholesterol synthesis-related genes (labeled in yellow) were identified from the REACTOME gene sets **(** **Table S1** **)**. The co-expression relations for pairwise combinations of these genes were investigated **(** **Figure 1B** **)**. CNV frequencies, represented by gain- and loss-of-function mutations, were inspected **(** **Figure 1C** **)**. SNVs were visualized by waterfall plots **(** **Figure 1D** **)**. A protein–protein interaction network was constructed **(** **Figure 1E** **)** and proteins were stratified into four clusters. Glycolysis-related genes were distributed in three clusters (Clusters I, II, and IV), whereas cholesterol synthesis-related genes were only assigned to Cluster III **(** **Table S2** **)**. Based on the GSVA scores for the glycolysis–cholesterol synthesis axis, a hierarchical cluster analysis was used to determine four metabolic subtypes of colon cancer: glycolytic, cholesterogenic, quiescent, and mixed **(** **Figure 1F** **)**. These novel subtypes could be used to distinguish among cases, as determined by a PCA **(** **Figure 1G** **)**.

### Relations between metabolic subtypes, clinical prognosis, and the tumor immune microenvironment

**Figure 2:**
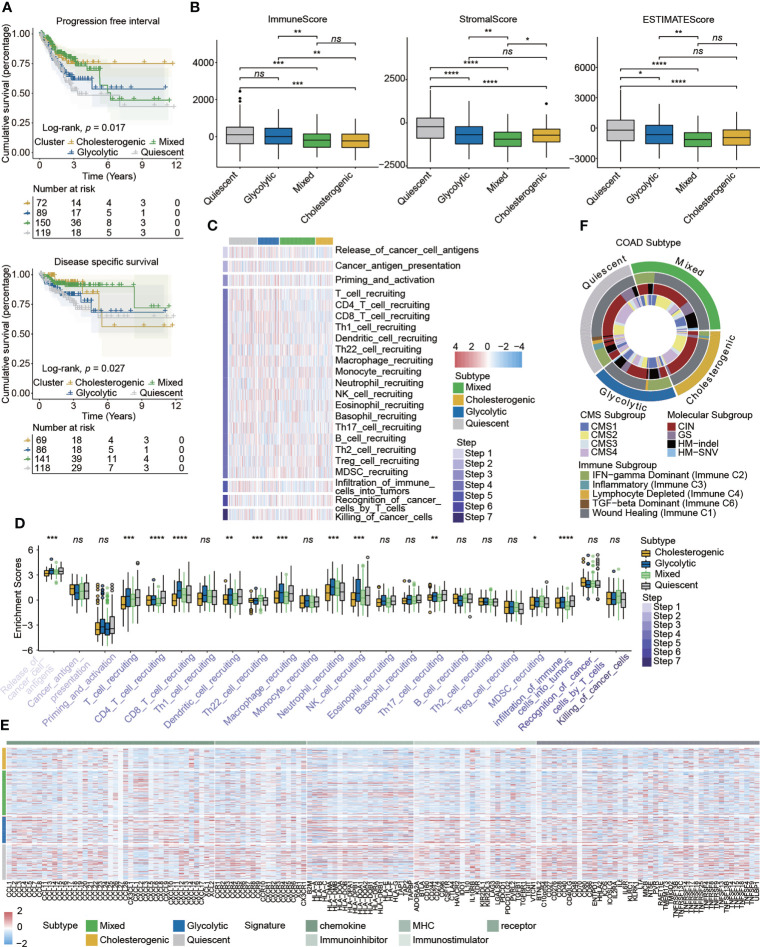
Correlations between metabolic subtypes and prognosis and the immune microenvironment in colon cancer. **(A)** Kaplan–Meier plots indicated that patients stratified into four metabolic subtypes differed with respect to the progression-free interval and disease-specific survival. **(B)** The tumor immune microenvironment was evaluated by the immune score, stromal score, and ESTIMATE score in different metabolic subtypes. **(C)** Heatmap detailing the activity of cancer–immunity cycle represented by seven steps. **(D)** Box plot showing the differences in cancer-immunity cycle steps among metabolic subgroups. **(E)** Heatmap detailing the associations between expression of immunomodulator signatures and metabolic subtypes. **(F)** Circus plot showing the relationship between the known molecular subtypes of colon cancer and metabolic subtypes. Statistical analysis: Wilcoxon rank-sum test. *ns*, nonsignificant, *p < 0.05, **p < 0.01, ***p < 0.001, ****p < 0.0001.

Although neither overall survival nor progression-free survival differed significantly among subtypes **(** **Figure S3** **)**, Kaplan–Meier survival analyses indicated that patients with different metabolic subtypes had different clinical prognoses evaluated by the progression-free interval (*p* = 0.017) and disease-specific survival (*p* = 0.027) **(** **Figure 2A** and **Table S3** **)**. The immune scores obtained by the ESTIMATE algorithm for glycolytic and quiescent cells were higher than those for cholesterol and mixed cells. The stromal score was higher in the quiescent group than in the other four subtypes, while the mixed subtype had a relatively low stromal score. There was no significant difference in stromal scores between the glycolytic and cholesterogenic groups. The results for ESTIMATE scores were consistent with those for stromal scores in comparisons among the four subtypes **(** **Figure 2B** **)**. A heatmap detailed the activity of the cancer–immunity cycle and suggested that most steps in this cycle were upregulated in glycolytic and quiescent cells and downregulated in cholesterol and mixed cells **(** **Figure 2C** **)**. A box plot illustrates that enrichment for functions related to the cancer immunity cycle differed among the metabolic subgroups **(** **Figure 2D** and **Table S4** **)**. Immunomodulators were upregulated in glycolytic and quiescent cells but downregulated in cholesterogenic and mixed cells **(** **Figure 2E** **)**. For instance, most immunomodulators, especially immune checkpoint targets (e.g., PDCD1, CD274, TIGIT, CTLA4, and IDO1), were upregulated in glycolysis. With respect to the signatures of chemokines, immune inhibitors, immune stimulators, MHC, and receptors, glycolytic and cholesterogenic subtypes were further compared **(** **Figure S4** **)**. The relationship between metabolic subtypes and other recognized molecular subgroups of colon cancer was also investigated. The cholesterogenic subtype contained an inflammatory subtype (immune C3), while the glycolytic subtype did not. The proportion of IFN-γ-dominant (Immune C2) cells in the glycolytic group was higher than that in the cholesterogenic group. The CMS subtype showed no clear relationship with the other three subtypes. Most cholesterogenic subtypes contained the CIN subtype, whereas most HM-indel subtypes were glycolytic and mixed. The GS subtype was mostly quiescent **(** **Figure 2F** **)**.

### Comparison of immunogenetic, genomic, and clinical parameters between glycolytic and cholesterogenic subgroups

**Figure 3:**
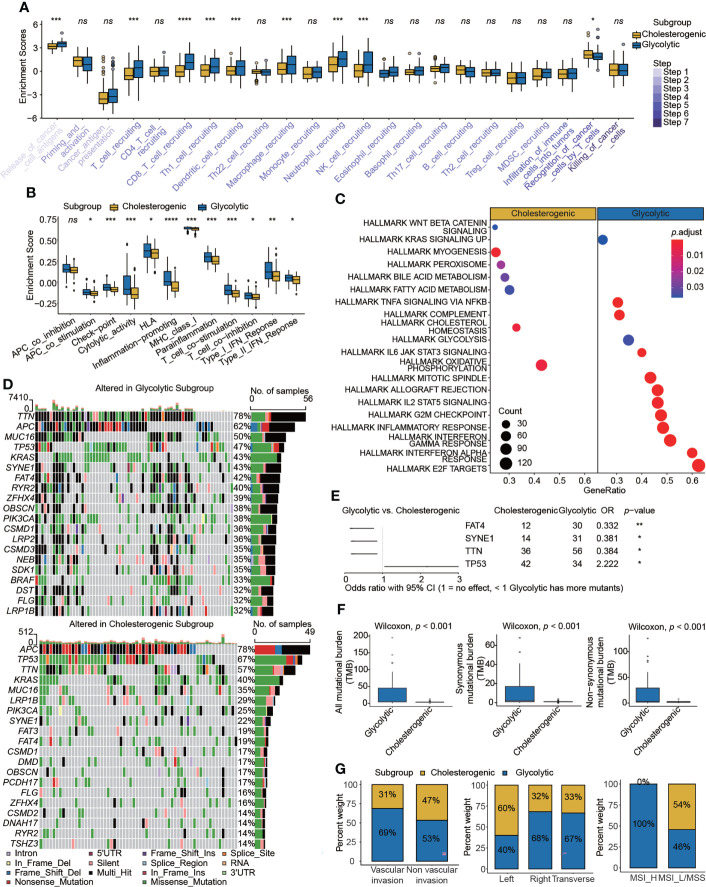
Comparison of immunogenetic, genomic, and clinical properties between glycolytic and cholesterogenic subgroups. Box plots showing differences in **(A)** cancer–immunity cycle and **(B)** immunotherapy signatures between the glycolytic and cholesterogenic subgroups. **(C)** Metabolism- and cancer-related pathways were enriched in a gene set enrichment analysis. **(D)** Waterfall plots displaying mutational landscapes. **(E)** Higher mutation frequencies of *FAT4, SYNE1*, and *TTN* were detected in glycolysis, whereas a high mutation frequency of *TP53* was detected in cholesterol. **(F)** Tumor mutational burden and **(G)** clinical characteristics, including vascular invasion, tumor site, and MSI status, in the two subgroups. Wilcoxon rank-sum test. *ns*, nonsignificant, *p < 0.05, **p < 0.01, ***p < 0.001, ****p < 0.0001.

The specific enrichment scores for the glycolytic and cholesterogenic subgroups evaluated by ssGSEA are shown in **Figure 3A** **(** **Table S5** **)**. We employed 12 immunotherapy-related gene sets and found that most were more highly activated in glycolysis than in cholesterol synthesis **(** **Figure 3B** **)**. To explore the biological functions of metabolic patterns in colon cancer, we performed a series of enrichment analyses of glycolytic and cholesterogenic pathways. A GO analysis showed significant differences between glycolytic and cholesterogenic factors with respect to neutrophil chemotaxis, neutrophil migration, chemokine activity, chemokine receptor binding, cytokine receptor binding, and cytokine activity, indicating that chemokines and cytokines were involved in this process **(** **Figure S5A** **)**. A KEGG analysis identified enrichment for various pathways, including cytokine–cytokine receiver interaction, toll-like receiver signaling pathway, natural killer cell-mediated cytotoxicity, IL-17 signaling pathway, and chemokine signaling pathway **(** **Figure S5B** **)**. The GSEA results showed that specific metabolism-related pathways were enriched **(** **Figure 3C** **)**. The mutational landscape in glycolytic and cholesterogenic pathways is shown in waterfall plots **(** **Figure 3D** **)**. *TTN, APC, MUC16, TP53*, and *KRAS* were the top five genes with the highest mutation frequencies. The mutation frequencies of *TTN, APC*, and *MUC16* in the glycolytic group were higher than those in the cholesterogenic group, whereas the mutation frequencies of *TP53* and *KRAS* were higher in patients in the cholesterogenic group. We compared genes with a mutation frequency of more than 30% and identified higher *FAT4, SYNE1*, and *TTN* mutation rates in glycolytic cells than in cholesterogenic cells, while a high *TP53* mutation frequency was detected in the latter group **(** **Figure 3E** **)**. The co-occurrence of three pairs mutations (*SYNE1-FAT4, TTN-FAT4*, and *SYNE1-TTN*) was observed the two metabolic subtypes. The co-occurrence of *SYNE1-FAT4* showed the most significant difference between groups (OR = 2.85, *p* < 0.01) **(** **Table S6** **)**. Glycolytic colon cancer exhibited a higher all mutational burden, synonymous mutational burden, and non-synonymous mutational burden **(** **Figure 3F** **)**. Patients stratified into the glycolytic subgroup exhibited distinct clinical characteristics, including vascular invasion, right and transverse colon cancer, and microsatellite instability (MSI)-high molecular features **(** **Figure 3G** **)**.

### Identifying hub genes involved in glycolysis and cholesterol synthesis

**Figure 4:**
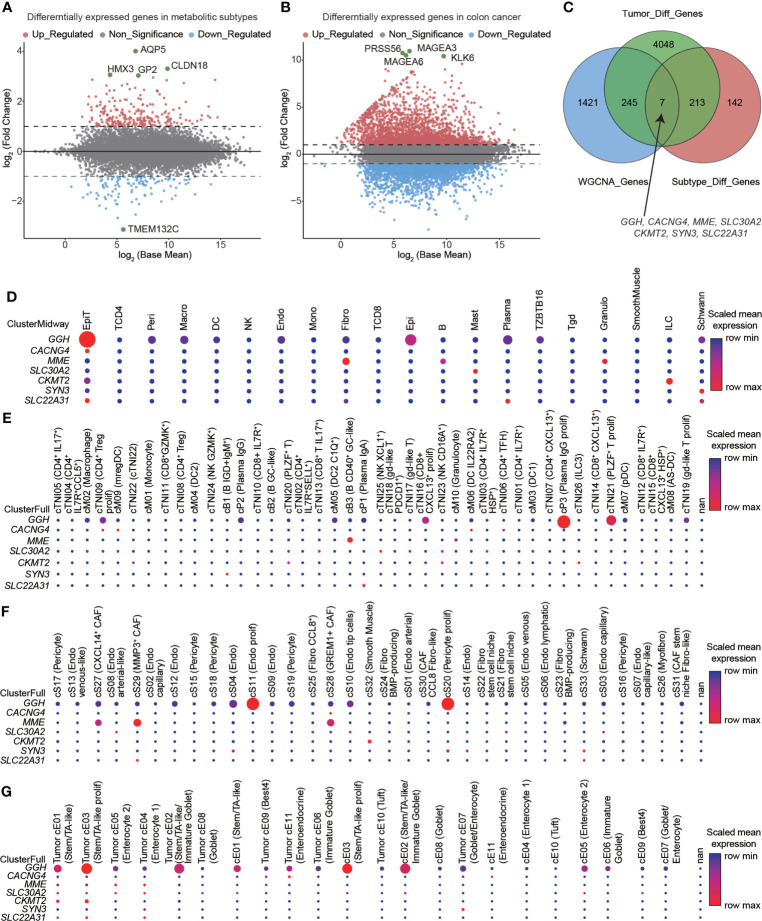
Hub genes identified in glycolytic and cholesterogenic pathways and the relationship between expression and the immune microenvironment. Volcano plots showing differentially expressed genes (DEGs) between **(A)** glycolytic and cholesterogenic groups and **(B)** colon cancer and normal tissues. **(C)** Venn diagram indicating the intersection of DEGs among metabolic subtype, colon cancer, and co-expressed gene modules. Seven hub genes were closely related to the functions of **(D)** immune cells, **(E)** immune cell subsets, **(F)** stromal cell subsets, and **(G)** epithelial cell subpopulations.

We used the RNA-seq count data from TCGA-COAD to identify DEGs in metabolic subtypes and colon cancer. We detected 362 DEGs (241 upregulated and 121 downregulated; **Figures 4A**, **S6A**) in glycolytic and cholesterogenic pathways and 4513 DEGs between colon cancers and normal samples, including 2443 upregulated and 2070 downregulated **(** **Figures 4B**, **S6B** **)**. We implemented WGCNA to identify co-expressed gene modules in metabolic subtypes. We determined six as the optimal soft threshold value **(** **Figure S7A** **)** and seven co-expression modules, excluding the MEgrey module with no co-expressed genes **(** **Figures S7B–D** **)** and identified MEblue as the key module (**Figure S7E**). We then took the intersection of the three sets and determined seven hub genes: *GGH, CACNG4, MME, SLC30A2, CKMT2, SYN3*, and *SLC22A31* **(** **Figure 4C** **)**. Evaluating scRNA-seq data, we found that the seven hub genes were closely related to the functions of immune cells **(** **Figure 4D** **)**, immune cell subsets **(** **Figure 4E** **)**, stromal cell subsets **(** **Figure 4F** **)**, and epithelial cell subsets **(** **Figure 4G** **)**. We employed methylation data from TCAG-COAD to analyze the relationship between the methylation status and transcript levels of seven hub genes. Five of the seven genes were within 200 bp upstream of the transcriptional start site, and their expression levels were significantly negatively correlated with methylation levels **(** **Figures S8A–E** **)**. The expression of *SLC30A2* had the strongest correlation with its methylation level (*r* = −0.54, *p* < 0.001). We investigated the expression levels of the seven hub genes in colon cancer-derived cell lines **(** **Figure S9** **)**.

### Clinical value of hub genes based on public colon cancer datasets

**Figure 5:**
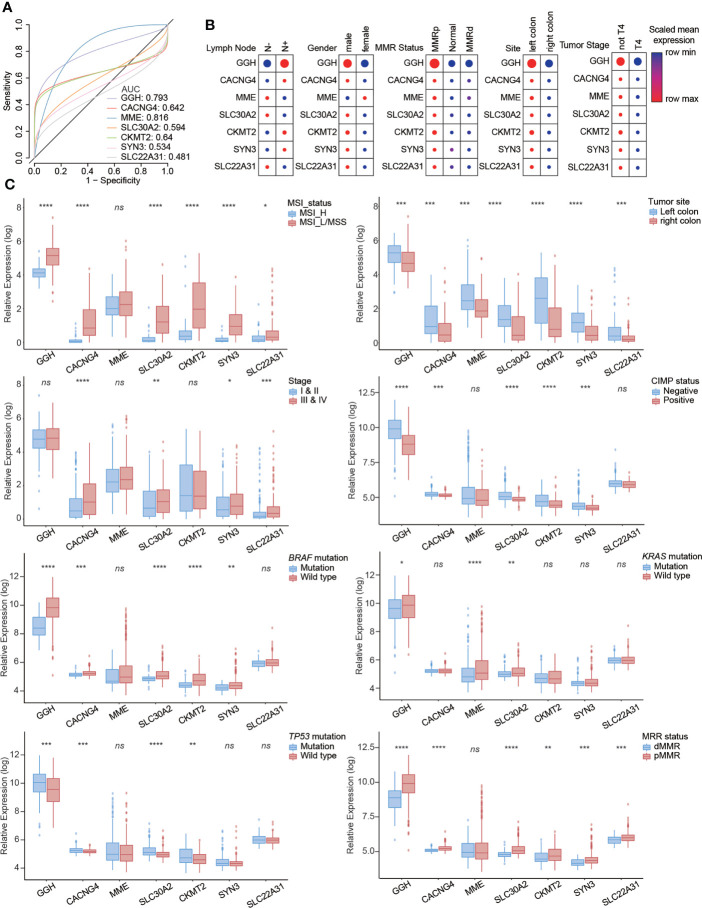
Clinical value of seven metabolism-related hub genes in colon cancer. **(A)** Diagnostic efficacy evaluated by receiver operator characteristic (ROC) curves. The relationships between hub genes and clinicopathological characteristics from **(B)** the single-cell dataset and **(C)** RNA-seq dataset. Statistical analysis: Wilcoxon rank-sum test. *ns*, nonsignificant, *p < 0.05, **p < 0.01, ***p < 0.001, ****p < 0.0001.

To explore the clinical value of seven metabolism-related hub genes, we utilized data from the single-cell dataset GSE178341, the microarray dataset GSE39582, and TCGA-COAD. The diagnostic efficacy of these hub genes was evaluated using receiver operating characteristic (ROC) curves. The areas under the curve (AUC) for GGH (0.793) and MME (0.816) were relatively high **(** **Figure 5A** and **Table S7** **).** The relationships between hub genes and clinicopathological characteristics were investigated **(** **Figures 5B, C**, **S10**), indicating that high GGH expression was related to clinicopathological characteristics, including MSI_L/MSS, left colon, negative CIMP status, wild-type BRAF and KRAS, TP53 mutation, and proficient mismatch repair (pMMR) status. We also performed a series of bivariate survival analyses combining hub genes with the clinical stage **(** **Figure S11** **)** and KRAS mutation status **(** **Figures S12**, **S13** **)**.

### Validating GGH expression and clinical implications in colon cancer tissue samples

**Figure 6:**
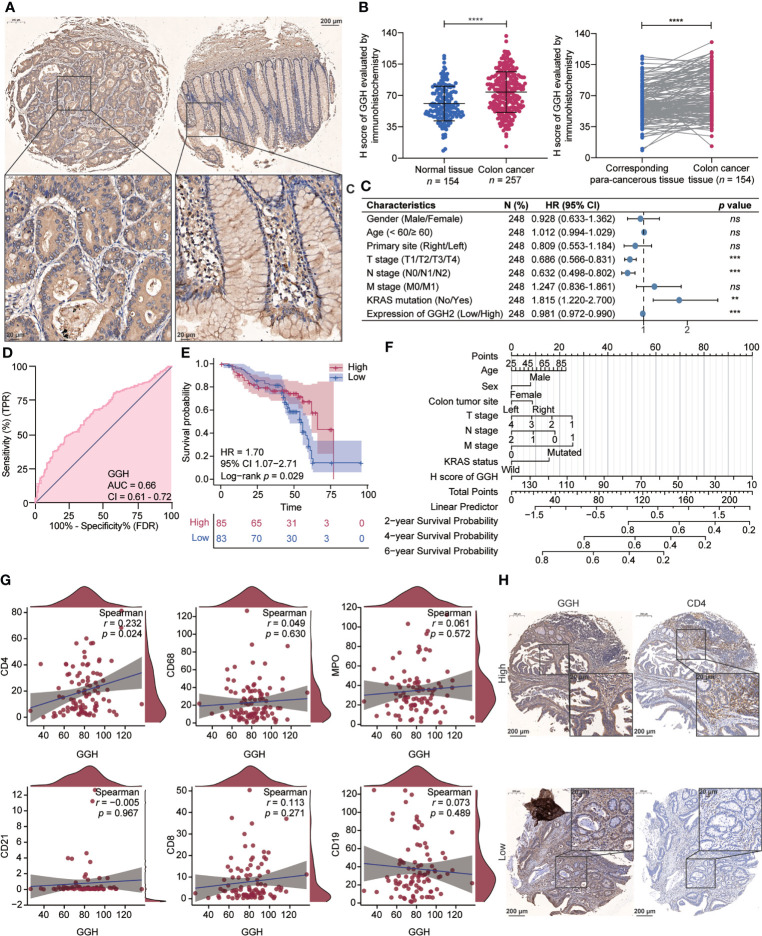
Gamma-Glutamyl Hydrolase (GGH) was differently expressed in colon cancer, and its high expression was related to tumor immune infiltration and clinical outcomes. **(A)** Representative images of GGH immunohistochemical staining in colon cancer and corresponding para-cancerous tissues. **(B)** GGH was more highly expressed in colon cancer tissues than in normal tissues (*p* < 0.001) or paired para-cancerous tissues (*p* < 0.001). **(C)** Forest plot showing that GGH expression is related to clinical characteristics. **(D)** ROC curve indicating the diagnostic efficacy of GGH in colon cancer. **(E)** Kaplan–Meier curves showed that patients with high GGH expression experienced favorable survival outcomes after surgery. **(F)** A nomogram based on the expression level of GGH was established to evaluate overall survival for each case. **(G)** Spearman’s correlation analyses were performed to evaluate the association between GGH expression and markers of tumor-infiltrating immune cells. **(H)** Immunohistochemical staining of consecutive sections indicated that GGH expression was positively correlated with CD4 expression. Scale bar = 20 or 200 μm. Statistical analysis: Mann–Whitney test and Wilcoxon rank-sum test. ****p < 0.0001.

Representative immunohistochemical staining results for GGH are displayed in **Figure 6A**. A quantitative analysis indicated that GGH was more highly expressed in colon cancer tissues than in normal tissues (*p* < 0.001) and paired para-cancerous tissues (*p* < 0.001, **Figure 6B**). GGH expression was significantly correlated with age (*p* = 0.006), T stage (*p* = 0.047), N stage (*p* = 0.014), KRAS mutation (*p* = 0.001), and survival status (*p* = 0.004) **(** **Table 2** **)**. Univariate and multivariate Cox regression analyses confirmed that high GGH expression (HR = 0.989, 95% CI 0.979–1.000, *p* = 0.041) was an independent prognostic factor **(** **Table 3**, **Figure 6C** **)**. The AUC of the ROC curve was 0.66 (CI: 0.61–0.72), supporting the diagnostic efficacy of GGH for colon cancer **(** **Figure 6D** **)**. Kaplan–Meier curves combined with the log-rank test revealed that patients (*n* = 168) with high GGH expression had a relatively longer overall survival (OS) after surgery (median 66 *vs*. 55 months, *p* < 0.05, **Figure 6E**). A nomogram, including clinical parameters and the H-score of GGH, was also established to evaluate the survival outcome for each case **(** **Figure 6F** **)**. Immunohistochemical staining results in consecutive sections indicated that the expression of GGH was correlated with markers of most infiltrating immune cells by Spearman correlation analyses, especially for CD4 (*r* = 0.230, *p* < 0.05, **Figure 6G**). Representative images are shown in **Figure 6H**.

**Table 2:** Correlation between GGH expression and clinical characteristics in 259 cases with successful IHC staining, analyzed by the chi-squared test.

Characteristics	GGH expression (Number)				*p* value
	Low	Percentage	High	Percentage
**Gender**					
Male	66	51.2%	80	61.5%	0.092
Female	63	48.8%	50	38.5%	
**Age**					
< 60	42	32.6%	64	49.2%	0.006
≥ 60	87	67.4%	66	50.8%	
**Primary colon cancer site**					
Right	56	43.4%	45	34.6%	0.147
Left	73	56.6%	85	65.4%	
**Radical surgery treatment**					
Yes	94	72.9%	83	63.8%	0.119
No	35	27.1%	47	36.2%	
**T stage**					
T1	10	7.8%	9	6.9%	0.047
T2	29	22.5%	49	37.7%	
T3	40	31.0%	37	28.5%	
T4	50	38.8%	35	26.9%	
**N stage**					
N0	25	19.4%	42	32.3%	0.014
N1	44	34.1%	48	36.9%	
N2	60	46.5%	40	30.8%	
**M stage**					
M0	94	72.9%	80	61.5%	0.052
M1	35	27.1%	50	38.5%	
**KRAS mutation**					
Yes	95	73.6%	39	30.0%	0.001
No	34	26.4%	91	70.0%	
**Survival status**					
Alive	56	43.4%	83	63.8%	0.004
Death	68	52.7%	41	31.5%	

**Table 3:** Relationships between GGH expression and clinical characteristics with overall survival were evaluated by univariate and multivariate Cox regression analyses.

Characteristics	Univariate Cox			Multivariate Cox		
	Hazard ratio	95% CI	*p* value	Hazard ratio	95% CI	*p* value
Gender	0.928	0.633 - 1.362	0.704			
Age	1.012	0.994 - 1.029	0.187			
Primary colon cancer site	0.809	0.553 - 1.184	0.276			
T stage	0.686	0.566 - 0.831	<0.001	0.917	0.599 - 1.404	0.690
N stage	0.632	0.498 - 0.802	<0.001	0.788	0.477 - 1.302	0.353
M stage	1.247	0.836 - 1.861	0.279			
KRAS mutation	1.815	1.220 - 2.700	0.003	1.374	0.881 - 2.143	0.162
Expression of GGH2	0.981	0.972 - 0.990	<0.001	0.989	0.979 - 1.000	0.041

### Confirming the inhibitory effects of GGH on glycolysis in colon cancer by cell and animal experiments

**Figure 7:**
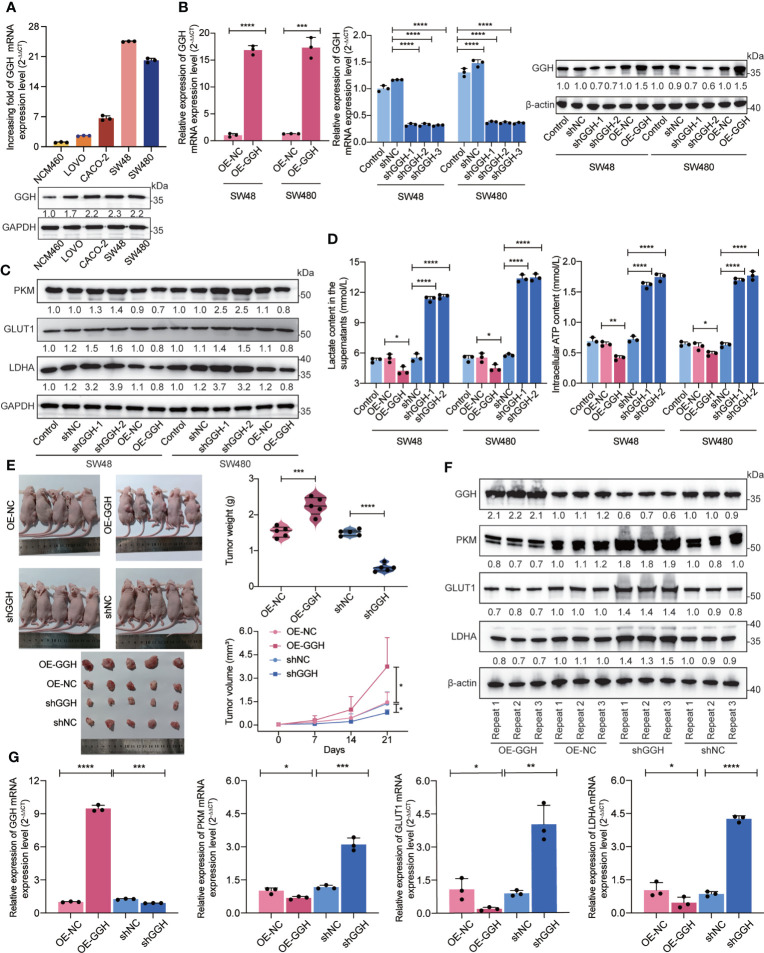
*In vitro* and *in vivo* experiments suggest that GGH inhibits glycolysis in colon cancer. **(A)** RT-qPCR and western blotting revealed that GGH at the mRNA and protein levels was more highly expressed in colon cancer-derived cell lines (LOVO, CACO-2, SW48, and SW480) than in normal colonic epithelial cells (NCM460). **(B)** GGH was stably overexpressed or silenced in SW48 and SW480 cells after transfection and selection. **(C)** The expression levels of PKM, GLUT1, and LDHA were assessed by western blotting. **(D)** The extracellular lactate content and intracellular ATP levels were detected by ELISA. **(E)** Subcutaneous tumor models were established to confirm the effect of GGH on colon cancer growth *in vivo*. Tumor volumes and weights were measured. **(F)** The mRNA levels of *GGH, PKM, GLUT1*, and *LDHA* in xenograft tumors were detected by RT-qPCR. **(G)** The protein levels of these biomarkers in xenograft tumors were detected by western blotting. β-Actin was used as an internal control. n = 3 biological replicates. Statistical analysis: Wilcoxon rank-sum test or Student’s *t*-test. *p < 0.05, **p < 0.01, ***p < 0.001, ****p < 0.0001.

The basal expression levels of GGH in a colonic epithelial cell line (NCM460) and colon cancer-derived cell lines (LOVO, CACO-2, SW48, and SW480) were detected using RT-qPCR and western blotting. As shown in **Figure 7A**, GGH was highly expressed in the colon cancer cell lines. GGH overexpression and interference efficiencies were also determined in the cDNA and RNAi groups **(** **Figure 7B** **)**. Based on previous bioinformatic results, we inspected the cardinal regulators of glycolysis, including PKM, GLUT1, and LDHA. These loci were downregulated in GGH-overexpressing cell lines and upregulated in RNAi-GGH cells, as determined by western blotting **(** **Figure 7C** **).** Additionally, GGH overexpression decreased the extracellular lactate content and intracellular ATP levels in cells, while the opposite results were obtained in siRNA-GGH cells using ELISA **(** **Figure 7D** **)**. To validate the effect of GGH in colon cancer, SW480-GGH and SW480-sh-GGH cells were subcutaneously injected into nude mice to establish xenograft models. Compared to tumors in the control group, smaller and larger tumors were visible on mice from the GGH overexpression and downregulation groups, respectively. GGH upregulation decreased the tumor volume and weight, while its downregulation had the opposite effect **(** **Figure 7E** **)**. Furthermore, the RNA **(** **Figure 7F** **)** and protein **(** **Figure 7G** **)** levels of GGH, PKM, GLUT1, and LDHA in each group were negatively regulated by GGH *in vivo*.

## Discussion

As metabolic reactions represented by glycolysis and cholesterol synthesis differ distinctly between colon cancer and healthy tissues at the transcriptional and protein levels according to enrichment analyses, we stratified cases into four subtypes: glycolytic, cholesterogenic, mixed, and quiescent. We determined that patients with different subtypes had different survival outcomes. Cui suggested that glycolysis-related genes are associated with a poor clinical prognosis in patients with colon cancer (30). In contrast, Jun demonstrated a reduction in a cholesterol synthesis rate-limiting enzyme, which facilitates the stemness and migration of colon cancer (31). We have previously demonstrated the prognostic value of several metabolic genes (32). Survival times in the glycolytic and cholesterogenic subgroups were relatively short and long, respectively, and these trends were particularly notable for progression-free interval and disease-specific survival.

Because metabolic interactions in the immune microenvironment trigger cancer progression (22), we evaluated the relationship between metabolic features and tumor immune cell infiltration. It is a paradox that certain metabolic subtypes with a high immune score (quiescent and glycolytic) exhibited worse survival outcomes than those of others with a high immune score (mixed and cholesterogenic). We also investigated correlations between the cancer–immunity cycle and the immunotherapy response (33) and revealed that steps, such as cancer cell antigen release, T cell recruitment, CD8 T cell recruitment, Th1 cell recruitment, dendritic cell recruitment, macrophage recruitment, neutrophil recruitment, and NK cell recruitment, were upregulated in glycolysis, while recognition of cancer cells by T cells was upregulated in the cholesterogenic group. That is, immune cells with high levels of infiltration did not function in glycolysis, while immune cells with low levels of infiltration functioned in cholesterol synthesis. We hypothesized that the glycolysis‒cholesterol synthesis axis primarily functions in the process of remodeling the immune microenvironment in colon cancer in tumor recognition by T cells, instead of immune cell recruitment. Cholesterol synthesis contributes to immune recognition and tumor death, whereas glycolysis plays an opposing role in recognition, resulting in recruitment failure (34, 35).

In contrast, chemokines and immunomodulators account for the activity of the cancer–immunity cycle, and immunological hallmarks of the tumor microenvironment include the expression of immunomodulators as well as inhibitory immune checkpoints, activity of the tumor immune cycle, and degree of infiltration of tumor-infiltrating immune cells (27). Renner reported that attenuating glycolysis in melanoma could augment checkpoint inhibitor responses (36). Ganapathy summarized the dysfunction of glycolysis in tumor-sensitized tumors in response to anticancer immunity (37). Kumagai found that lactate upregulates PD-1 expression in CD8+ T cells and regulatory T cells in a highly glycolytic tumor environment (38). Our analyses suggested that a high level of immune infiltration leads to the upregulation of immunomodulators in glycolysis, suggesting that immunotherapeutics promoting tumor cell recognition by T cells are promising.

As a crucial driver of colon cancer development, KRAS-related signaling is enriched in glycolysis. Ying demonstrated that KRAS regulates anabolic glucose metabolism *in vivo* (39). Wong et al. reported that mutant KRAS-expressing colon-derived cell lines are dependent on glutamate-based glycolysis (40). In this study, we evaluated the prognostic value of KRAS mutations and identified key genes involved in glycolysis. Liu reported that colon cancer features DNA hypermethylation and mutations in KRAS and can be stratified into genome stable (GS) subgroups (28). We compared the novel metabolic subtypes with these previously established subgroups (6, 28, 29) and found that GS was more closely related to the glycolytic than the cholesterogenic subtype.

We surveyed the mutational landscapes of metabolic subtypes and identified differences in the co-occurrence of *SYNE1-FAT4* mutations among metabolic subtypes. *SYNE1* encodes a multi-isomeric modular protein that forms a linking network and maintains subcellular localization. *SYNE1* mutations are associated with cerebellar recessive ataxia and bipolar disorder (41, 42). *FAT4* encodes calcium-dependent cell adhesion proteins, and its genetic variants are involved in recessive syndromes and periventricular neuronal heterotopia (43). We investigated the clinicopathological characteristics of the glycolytic and cholesterogenic subtypes. Because patients with a high level of TMB or MSI-high colon cancer achieve a favorable checkpoint inhibitor response (5, 44), we speculate that patients in the glycolytic group are more sensitive to immunotherapy than are those in the cholesterogenic group, consistent with previous analyses of the immune microenvironment. The glycolytic subtype displayed more adverse pathological prognostic features, such as vascular invasion, right and transverse colon cancer, than those in the cholesterogenic subtype, and such features are correlated with poor survival outcomes (4).

Seven hub genes were closely correlated with immune cells with different characteristics. GGH showed a closer association with a variety of immune cells, immune cell subsets, and stromal cell subsets than with others, including EpiT, macrophages, PLZF^+^ T, endothelial cells, pericytes, and epithelial subpopulations. Furthermore, GGH expression was positively related to the infiltration of CD4^+^ T cells in colon cancer in clinical samples. Schmee found that *Helicobacter pylori* produced gamma-glutamyl transpeptidase, which could inhibit CD4^+^ T cell proliferation and infiltration in the gastric mucosa, resulting in the development of peptic ulcer disease, gastric adenocarcinoma, and even mucosa-associated lymphoid tissue lymphoma (45). Instead, γ-glutamyl hydrolase catalyzes the hydrolysis of folyl-poly-γ-glutamates and anti-folyl-poly-γ-glutamates to produce folic acid and glutamate. γ-Glutamyl hydrolase has been identified as a prognostic biomarker for malignant tumors (46, 47) and a predictive biomarker of fluorouracil-based chemotherapy regimens for gastrointestinal cancer, as it regulates folate metabolism (48–50). Although our study preliminarily demonstrated that GGH repressed glycolysis, which has been considered to be driven by KRAS mutations in malignant tumors (51, 52), the mechanism by which GGH exerts its impact on tumor immune cell infiltration or the anti-tumor immune response vie the regulation of metabolic reactions or other crucial gene expression requires further exploration.

Our study had some limitations. Our bioinformatic analyses provide a preliminary overview of connections between metabolic reprogramming in tumor cells and the tumor microenvironment, and further studies are needed to determine the mechanisms by which the metabolic reprogramming of specific cells, such as immune cells, fibroblasts, and adipocytes, impact the tumor microenvironment. Bioinformatic analyses provide insights into the differences in the immune microenvironment of colon cancer based on metabolic subgroups, and the connections should be verified experimentally. The prognostic or predictive value of glycolysis‒cholesterol synthesis subtypes in colon cancer should be validated in large clinical trials with retrospective and prospective cohorts. The specific molecular mechanisms by which GGH functions in metabolism and CD4^+^ T cell immune infiltration also require verification by laboratory experiments.

## Data availability statement

All data used in this work can be acquired from the GDC portal (https://portal.gdc.cancer.gov/), the International Cancer Genome Consortium (ICGC, https://dcc.icgc.org/) and the Gene- Expression Omnibus under the accession number GSE178341 and GSE39582.

## Ethics statement

The studies involving human participants were reviewed and approved by the Ethics Committee of Zhongshan Hospital, Fudan University (ethical approval number: B2020-168R). The patients/participants provided their written informed consent to participate in this study. Xenograft experiments were performed with the approval of the Animal Experiments Ethics Committee of Zhongshan Hospital, Fudan University.

## Author contributions

LL conceived the study. T-SL designed the methods and experiments. X-ZS performed the data curation and manuscript revision. LL and Y-JC undertook the bioinformatic analysis and drafted the manuscript. Y-JC and XG carried out the cell and animal experiments. XG and M-LL were responsible for immunohistochemical analysis. M-LL assisted in the experiments. Y-YY and Y-HC supplied clinical data.
