# Supplementary figures and images for: Interaction between glycolysis‒cholesterol synthesis axis and tumor microenvironment reveal that gamma-glutamyl hydrolase suppresses glycolysis in colon cancer
Source: Front Immunol. 2022 Dec 7;13:979521. doi: 10.3389/fimmu.2022.979521 (PMC9767965; doi:10.3389/fimmu.2022.979521)

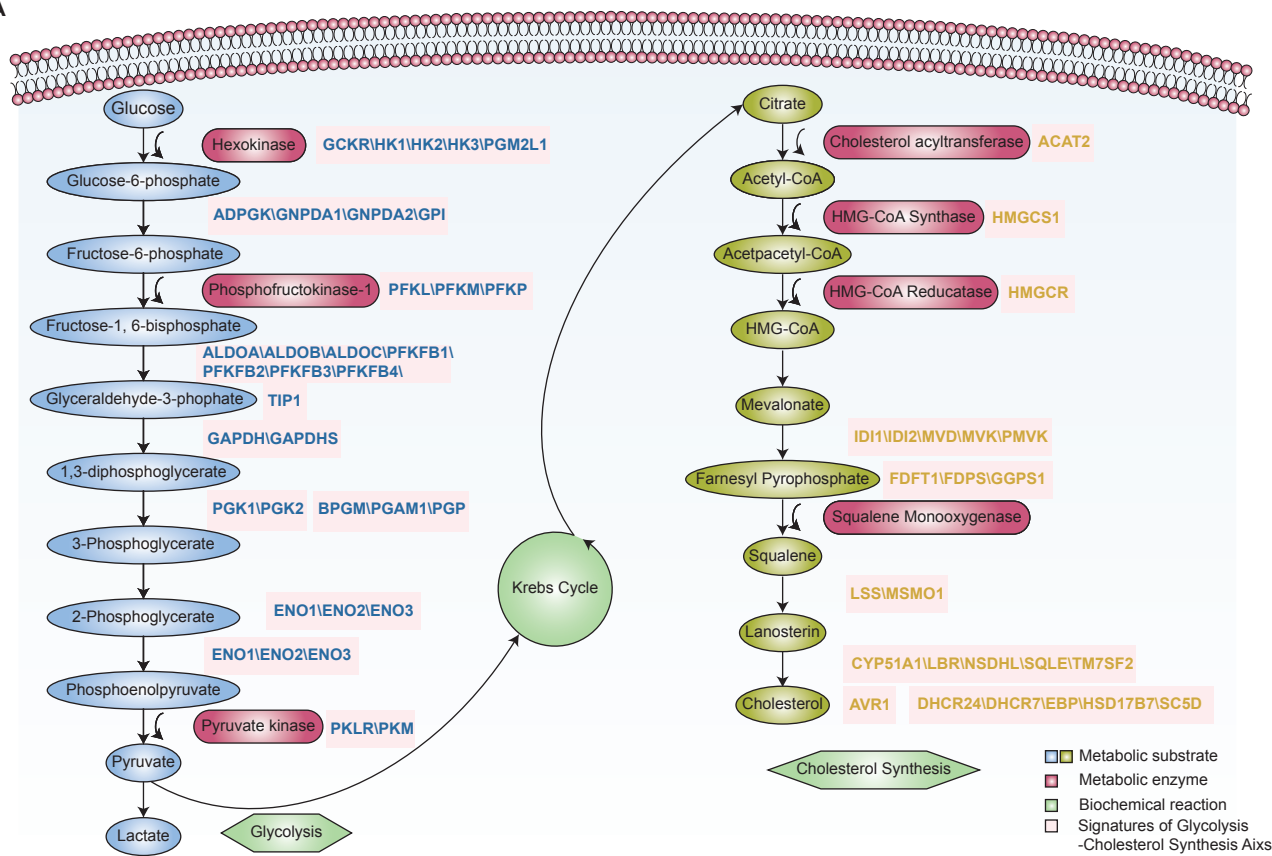

**B**

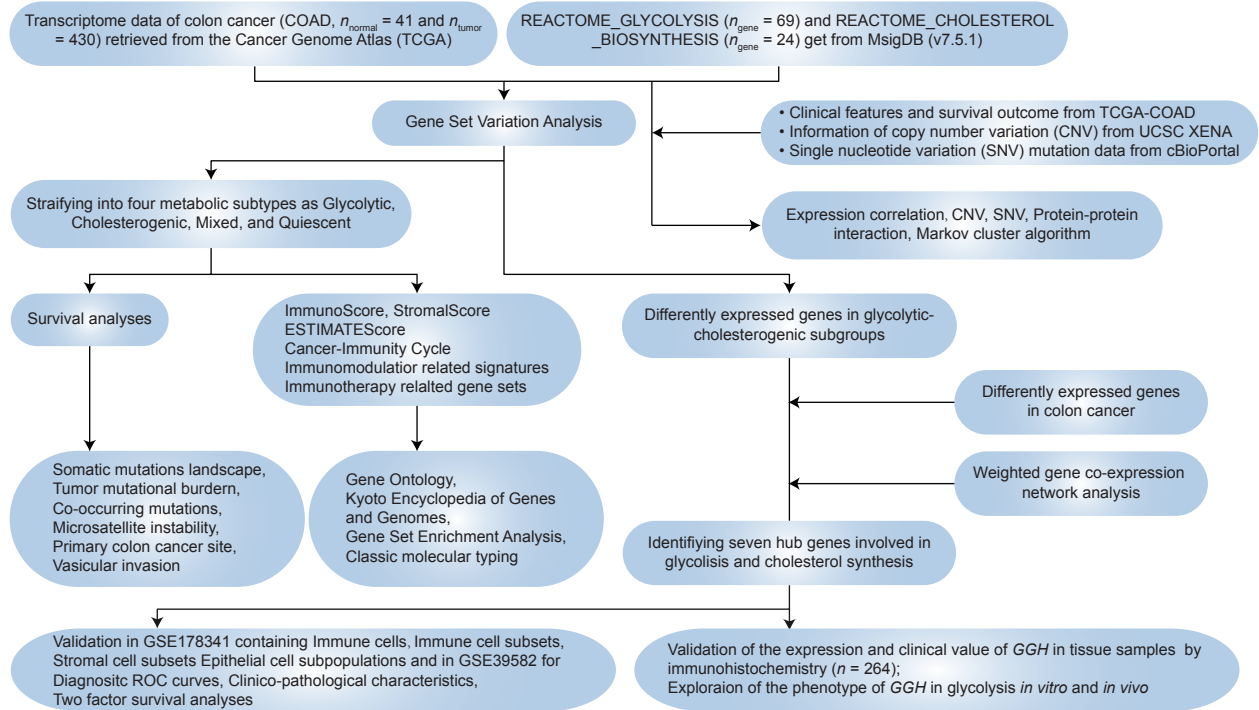

Supplement: Supplementary file 1 [file Image_1.pdf]

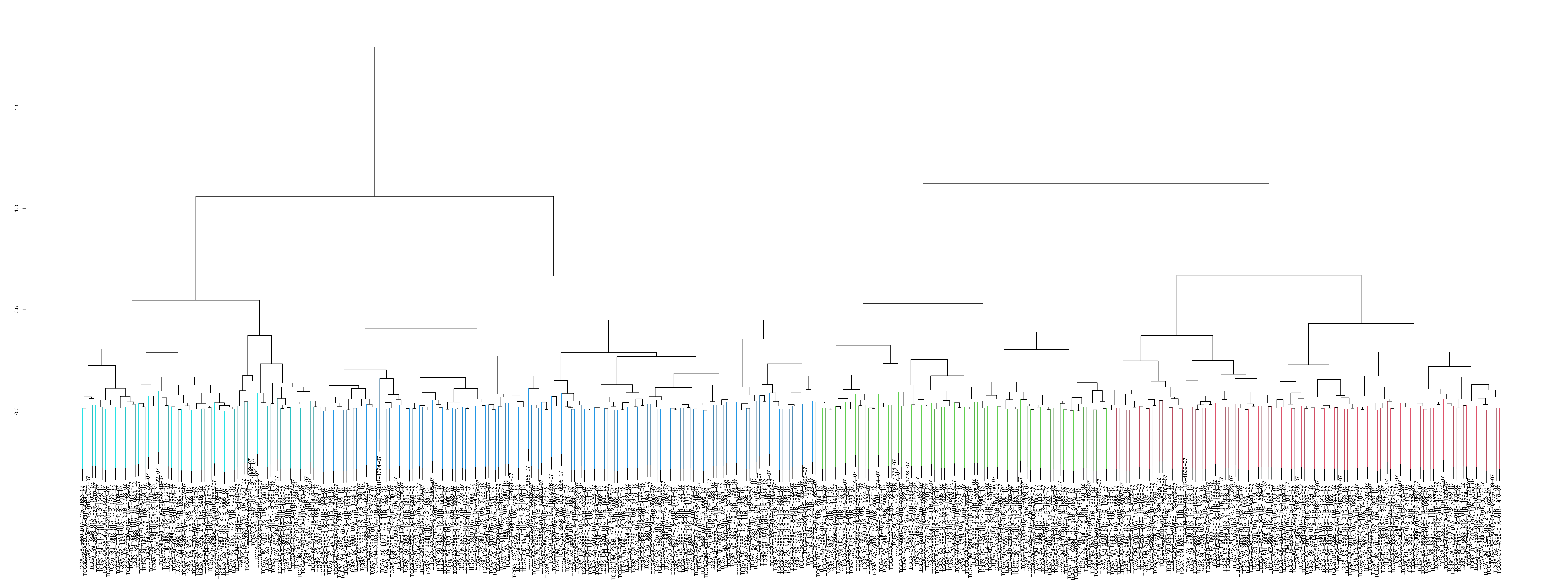

Supplement: Supplementary file 2 [file Image_2.pdf]

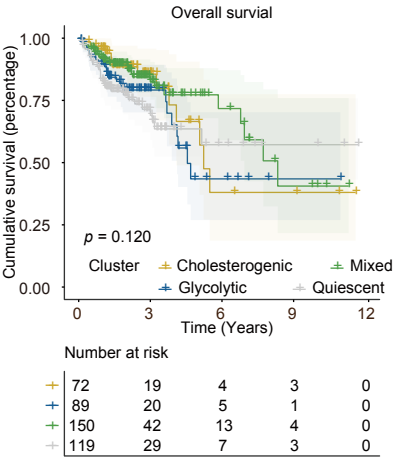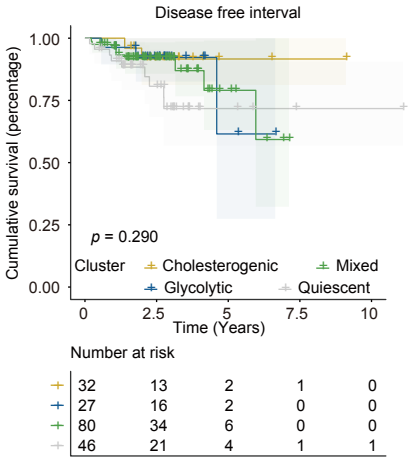

Supplement: Supplementary file 3 [file Image_3.pdf]

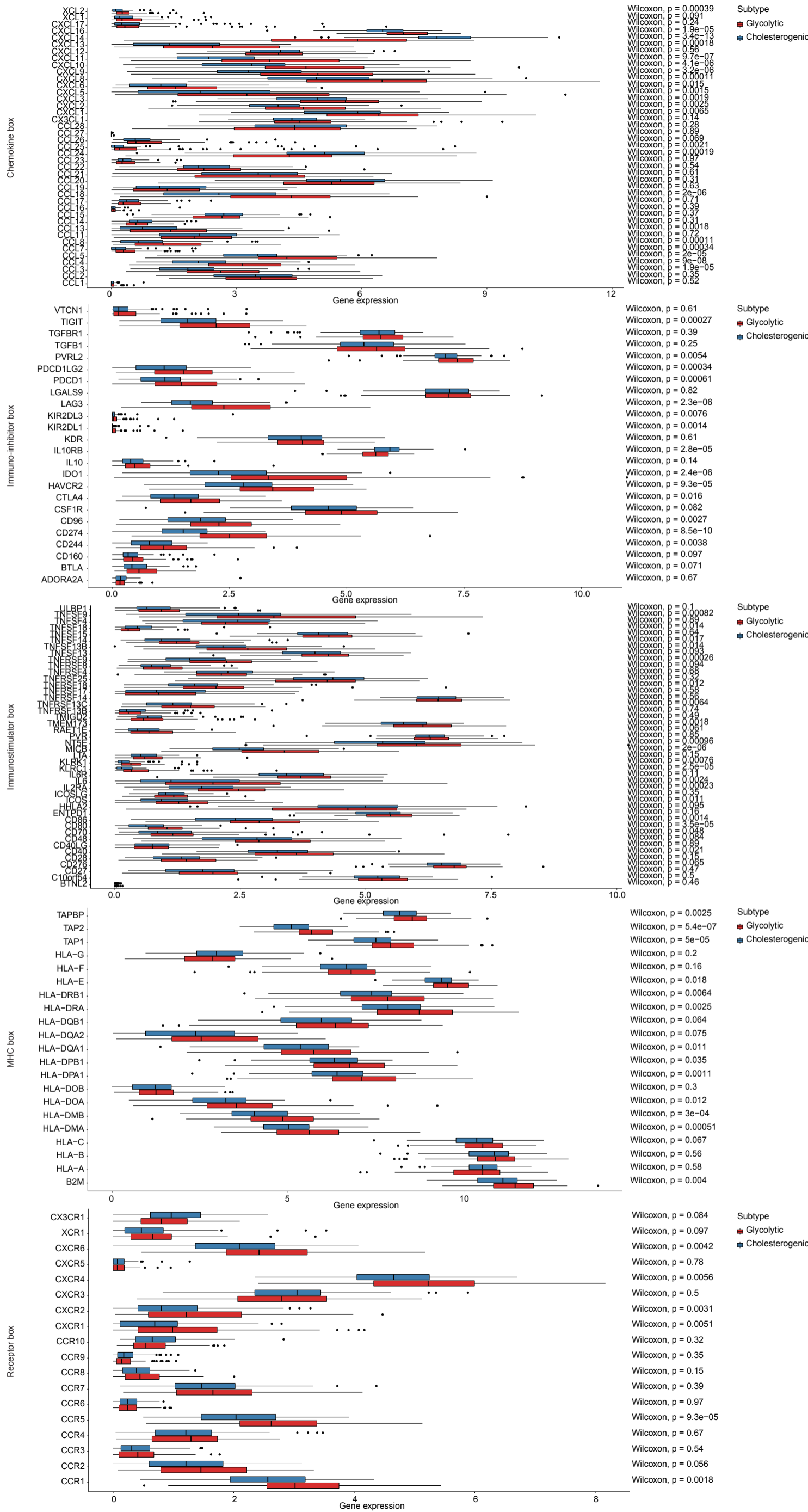

Supplement: Supplementary file 4 [file Image_4.pdf]

**A**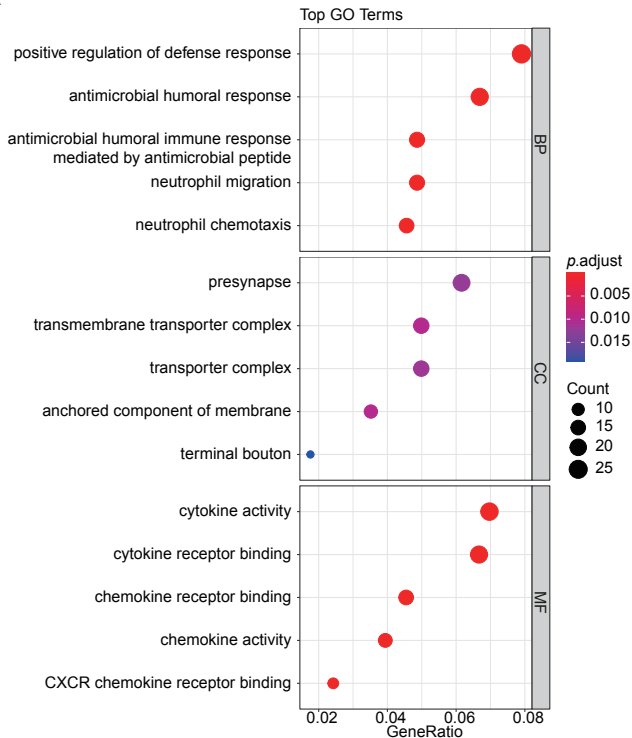**B**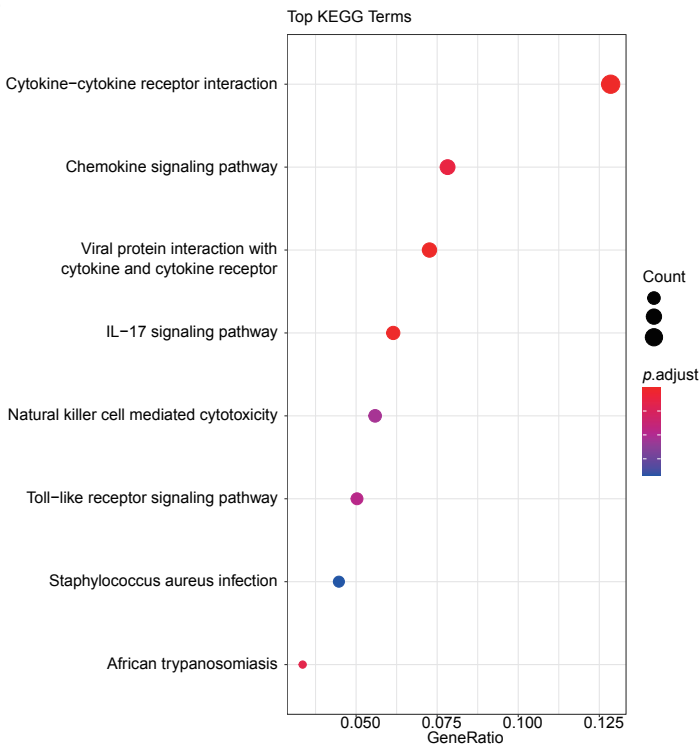

Supplement: Supplementary file 5 [file Image_5.pdf]

A

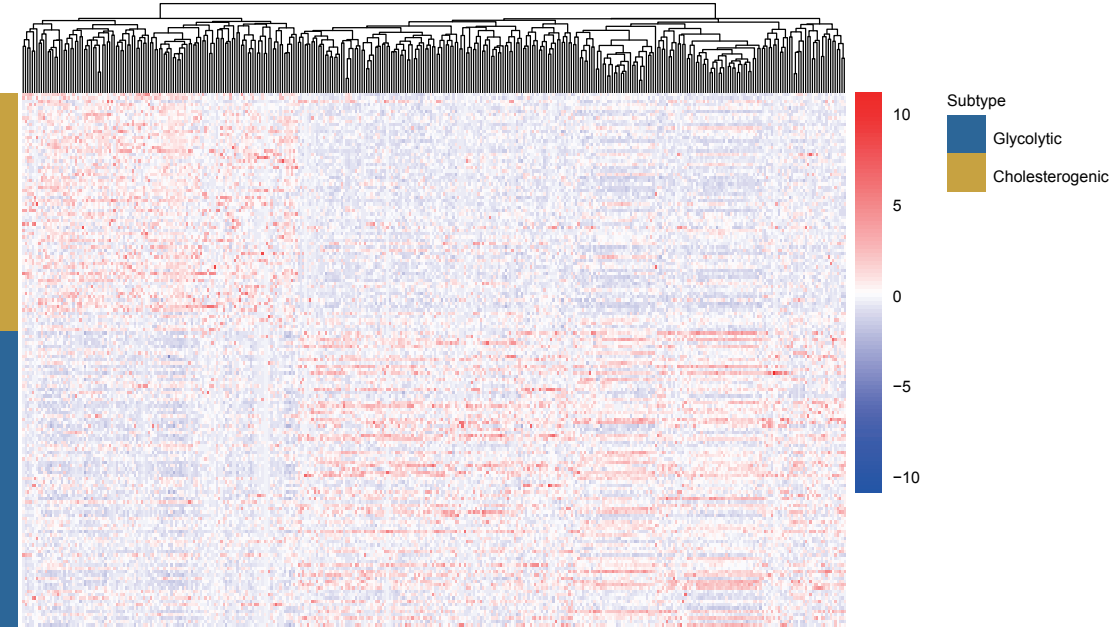

B

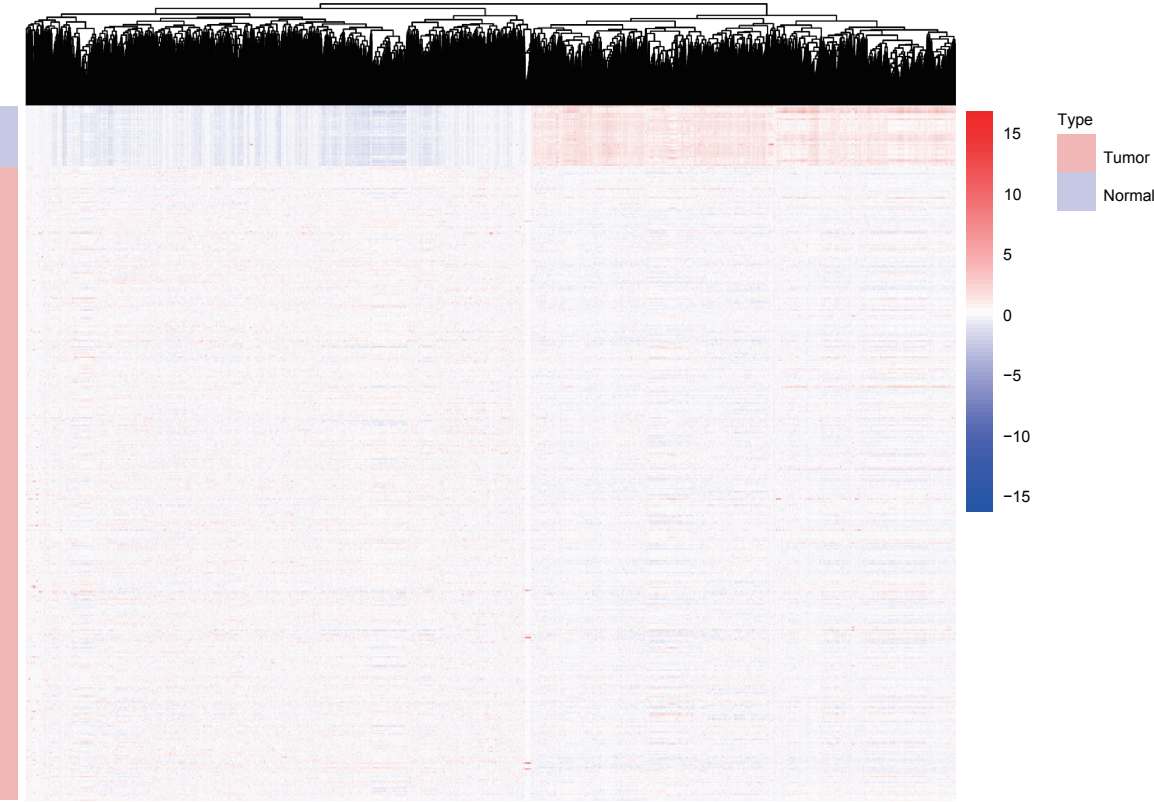

Supplement: Supplementary file 6 [file Image_6.pdf]

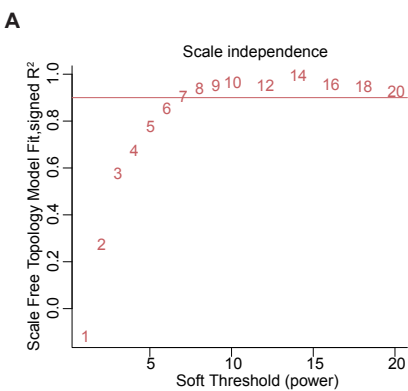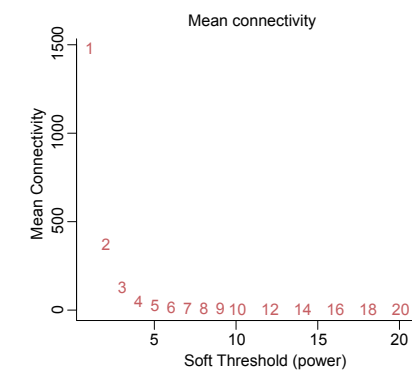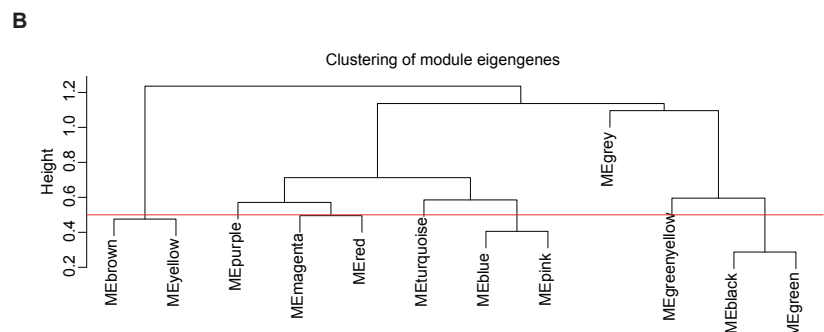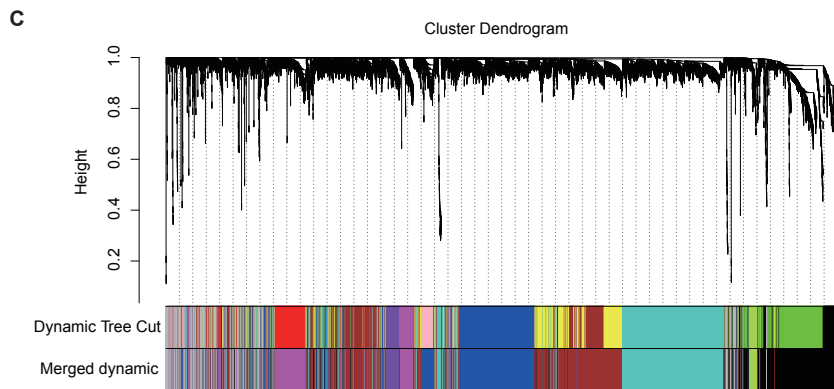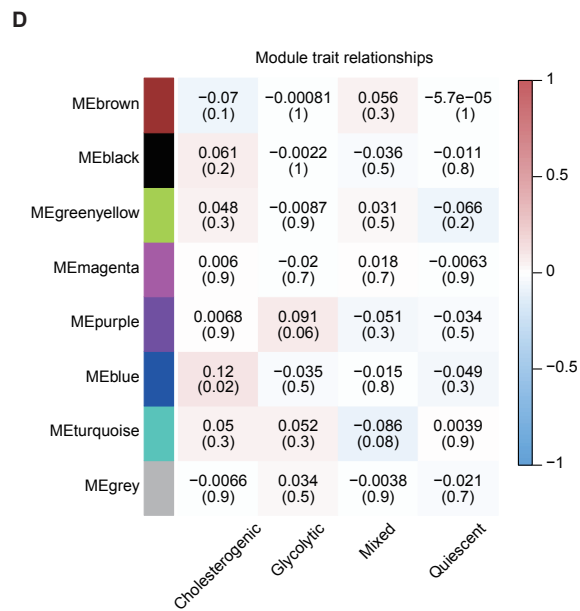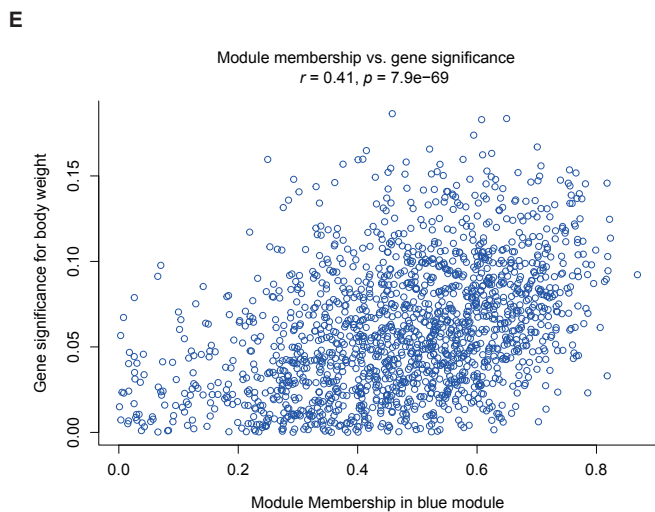

Supplement: Supplementary file 7 [file Image_7.pdf]

**A**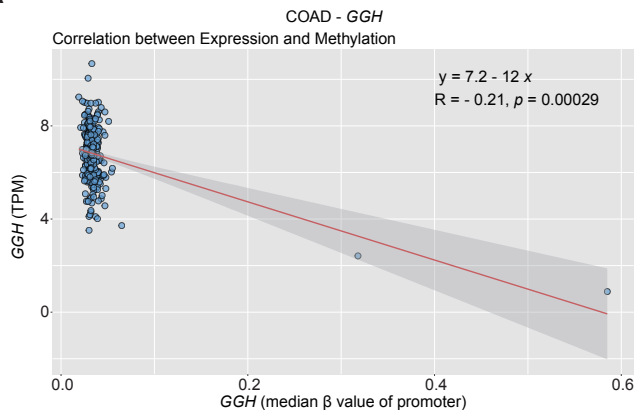**B**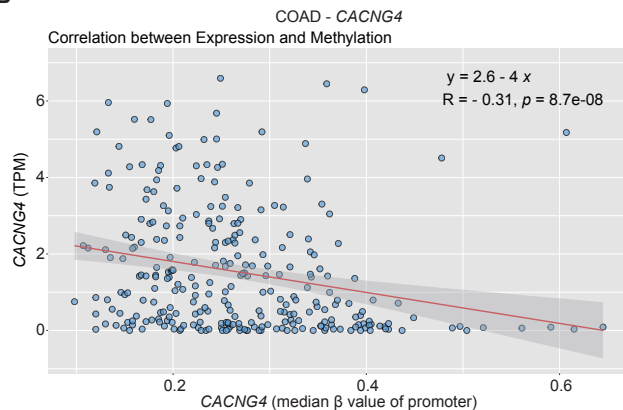**C**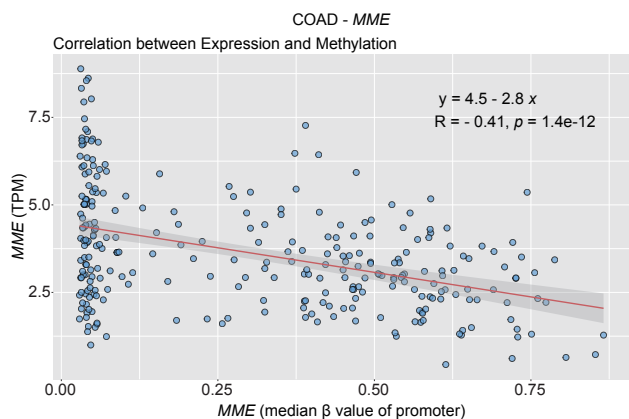**D**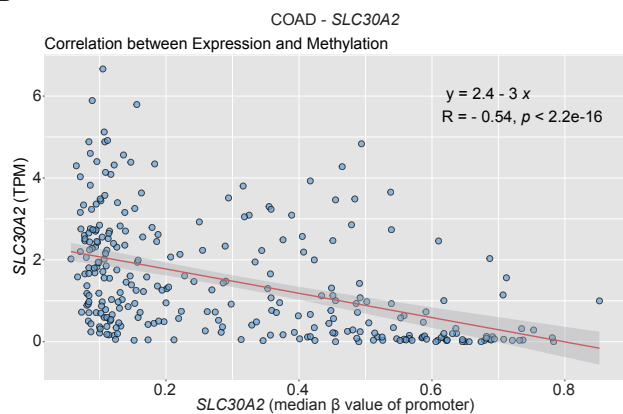**E**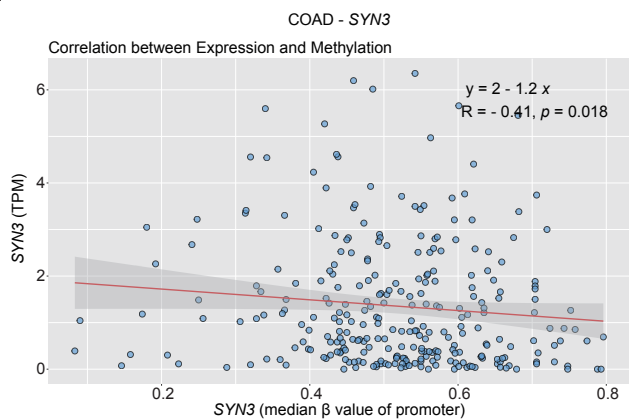

Supplement: Supplementary file 8 [file Image_8.pdf]

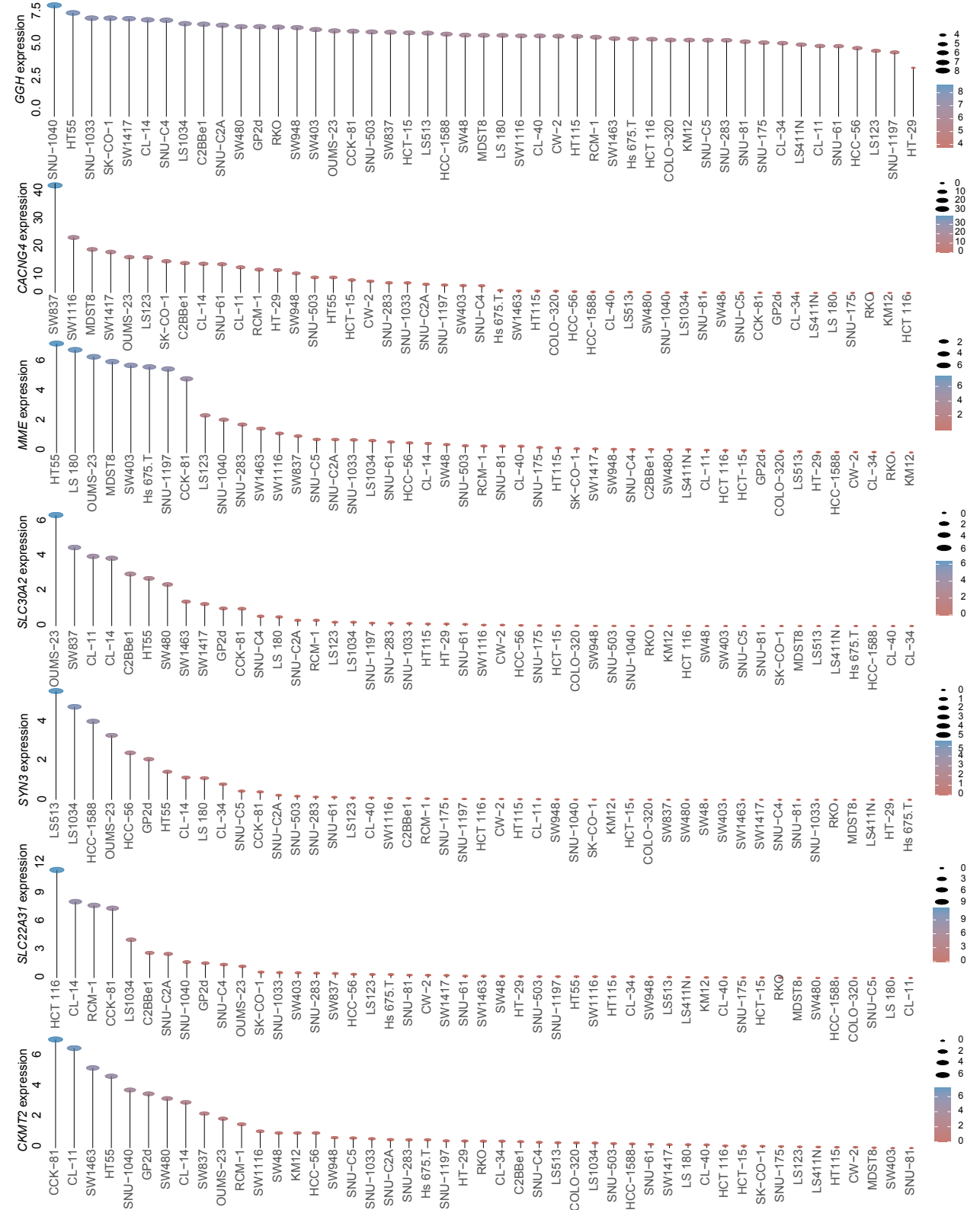

Supplement: Supplementary file 9 [file Image_9.pdf]

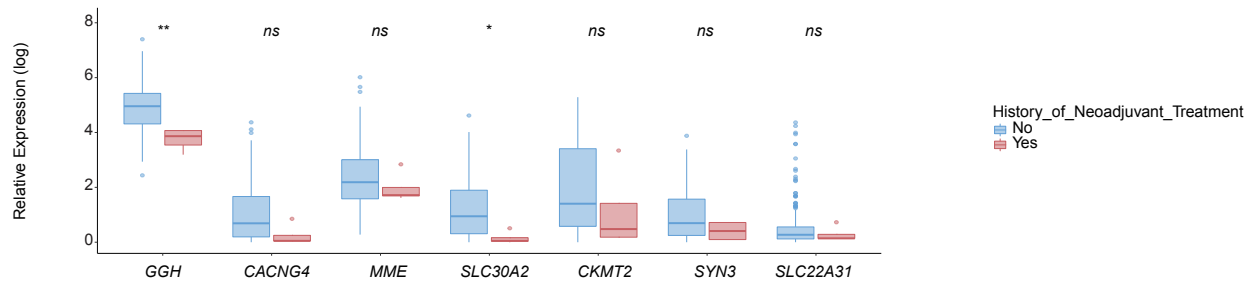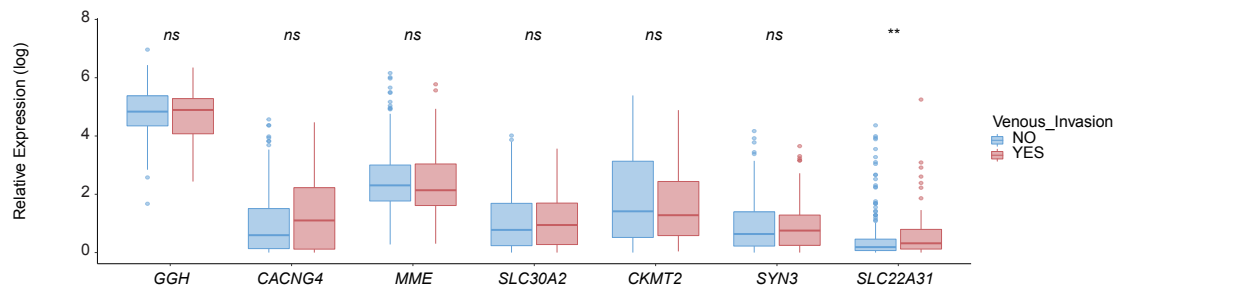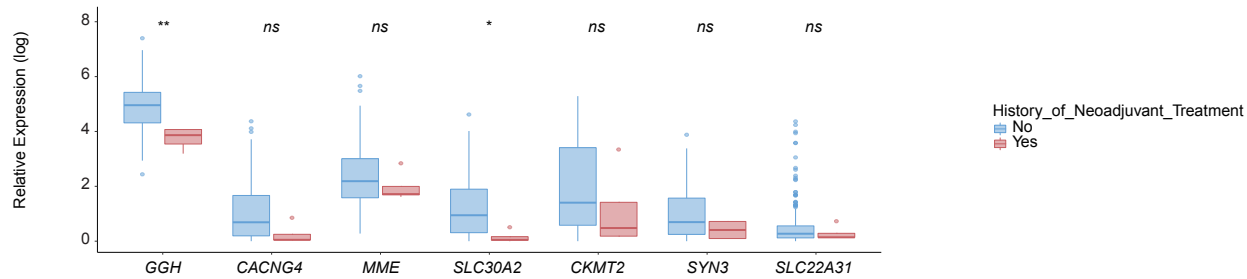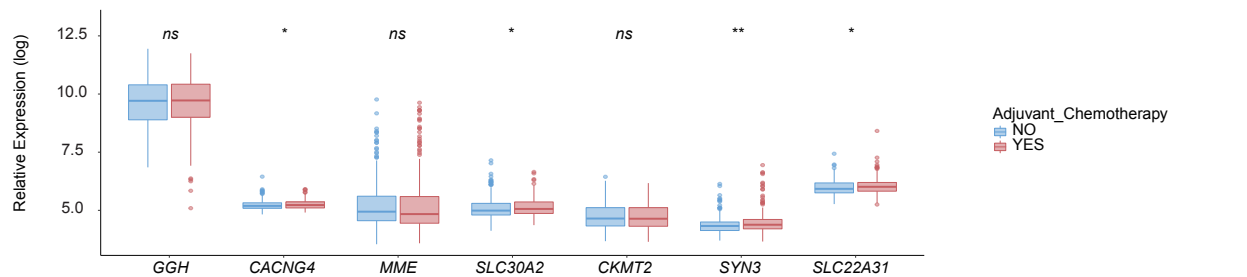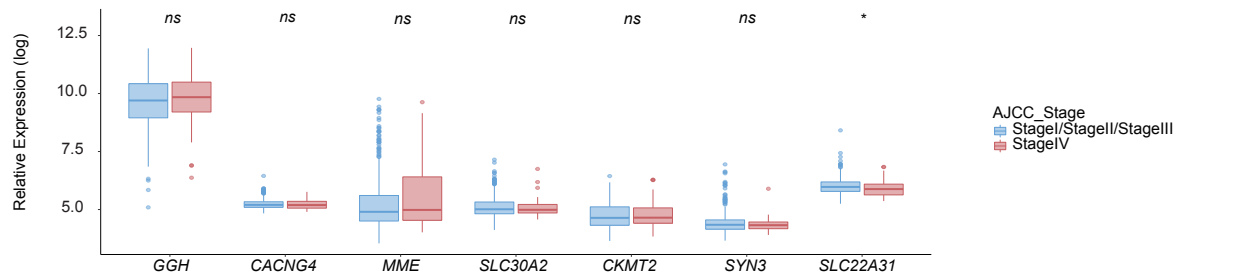

Supplement: Supplementary file 10 [file Image_10.pdf]

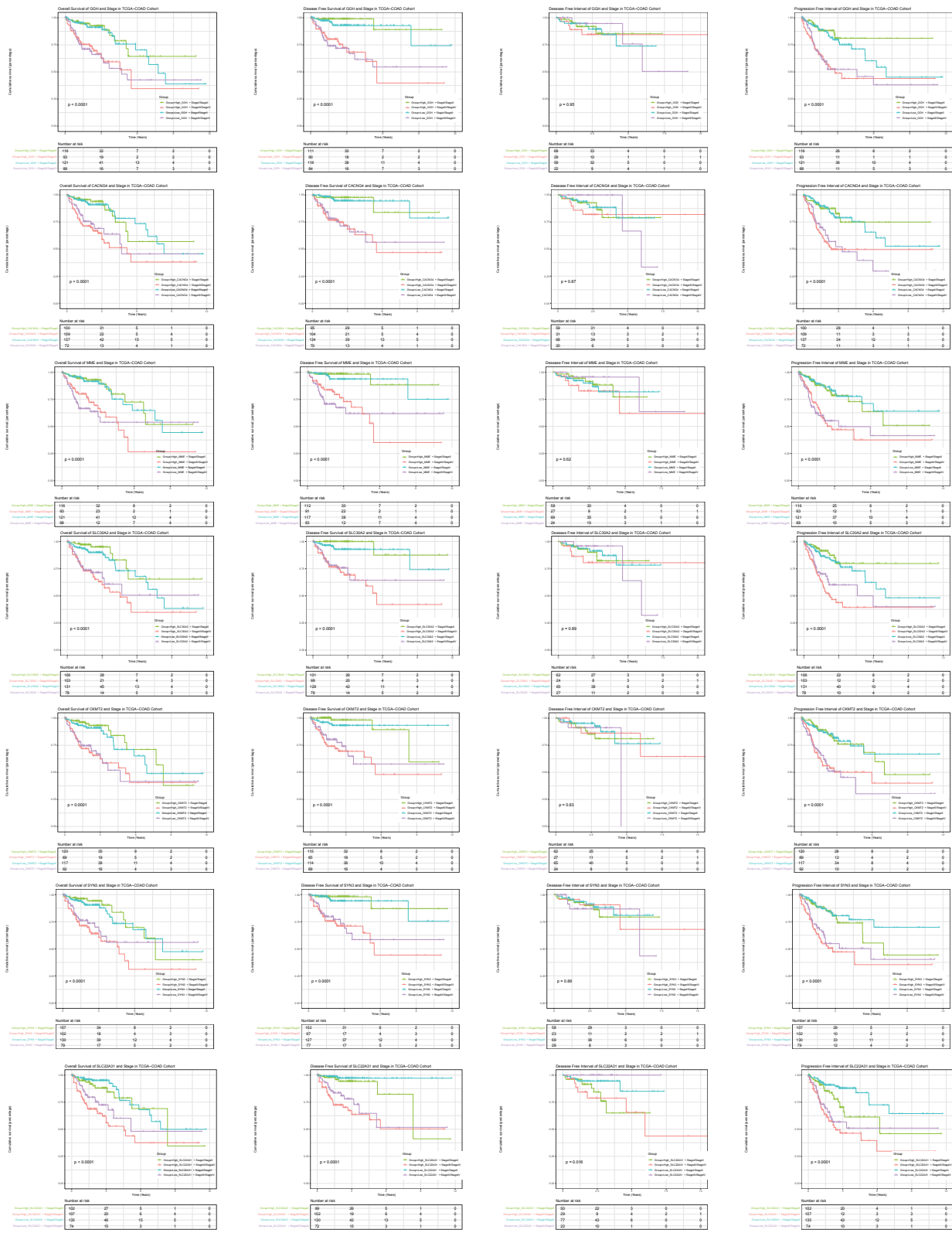

Supplement: Supplementary file 11 [file Image_11.pdf]

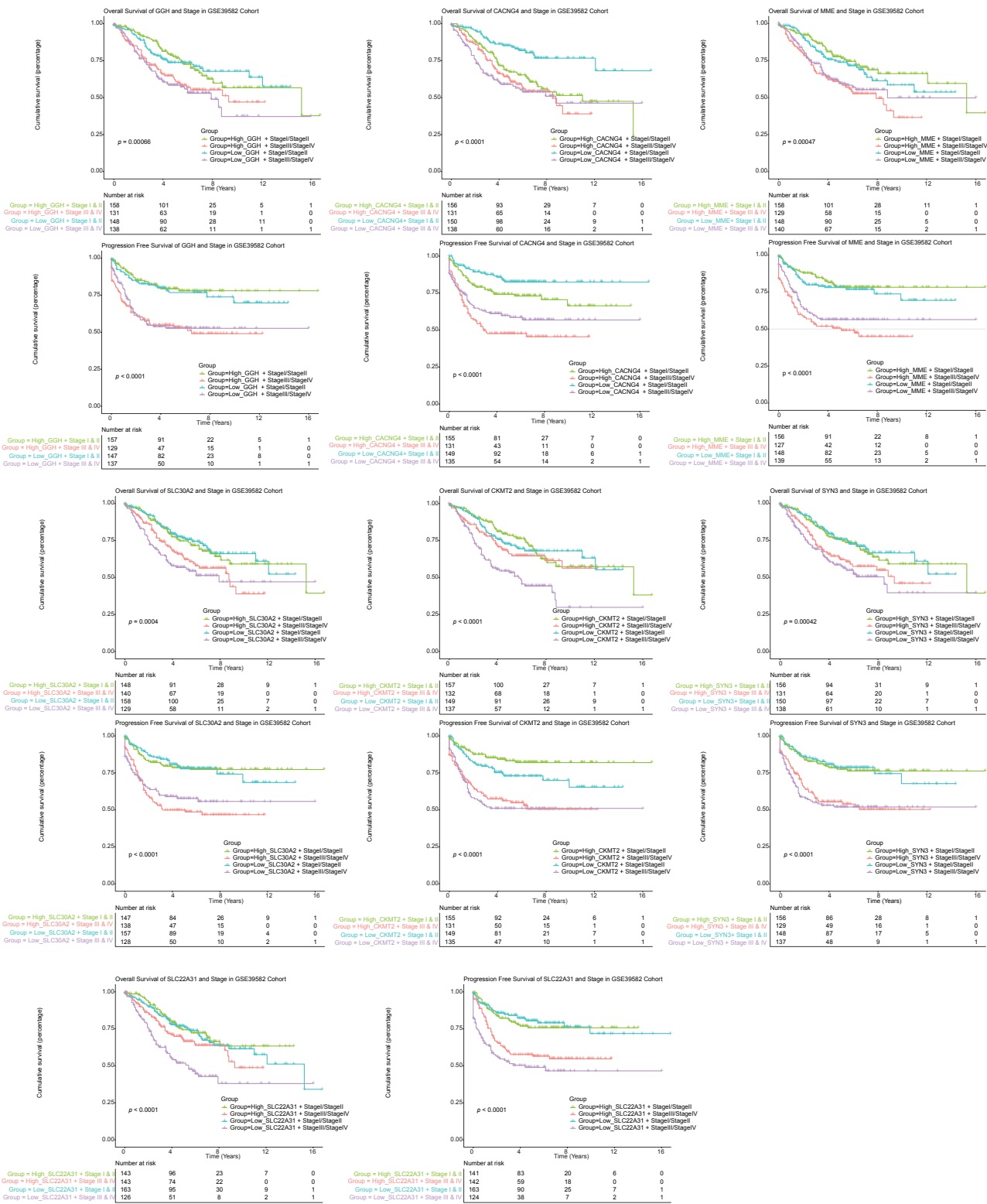

Supplement: Supplementary file 12 [file Image_12.pdf]

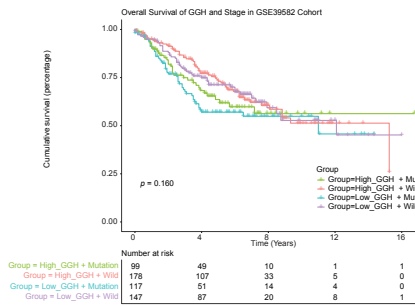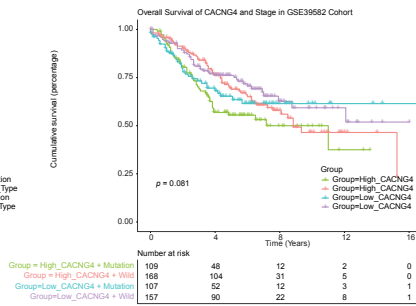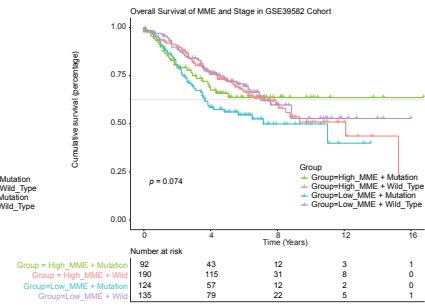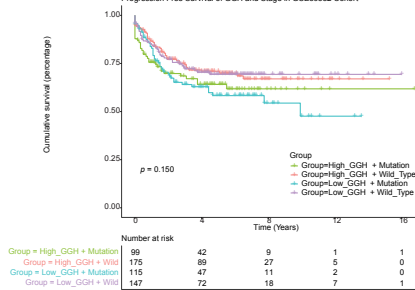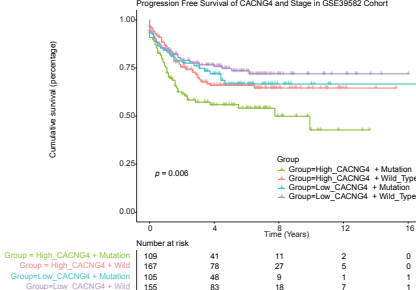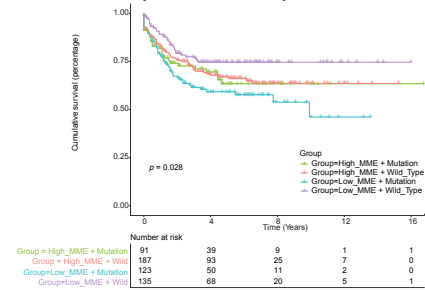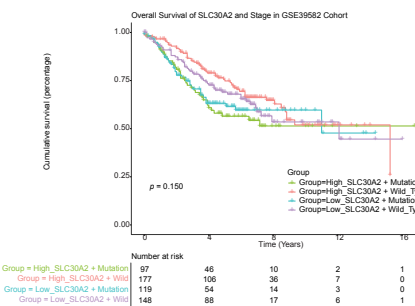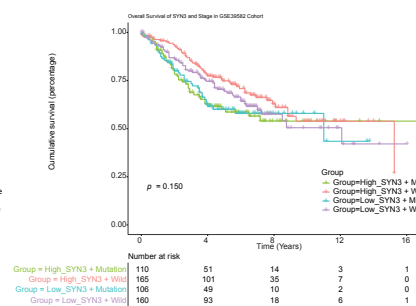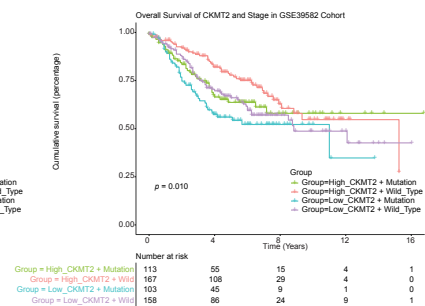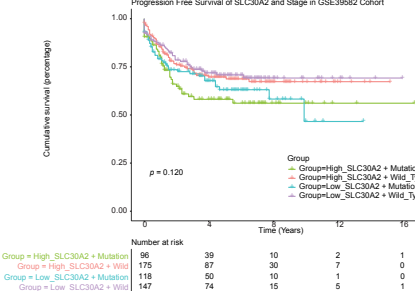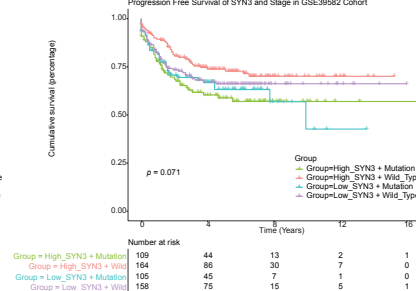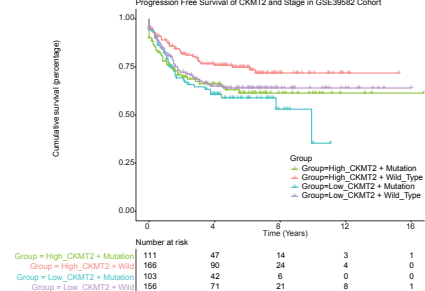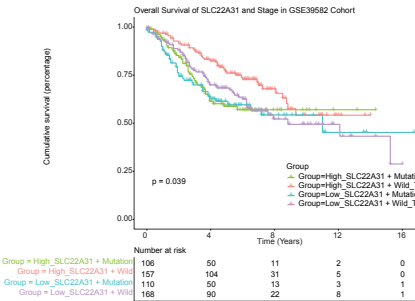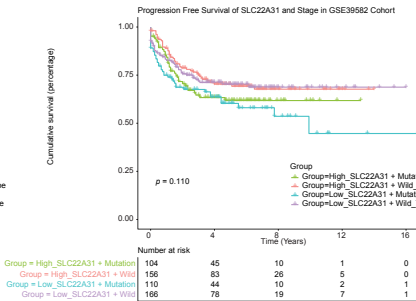

Supplement: Supplementary file 13 [file Image_13.pdf]
