# Supplementary material for: Interaction between glycolysis‒cholesterol synthesis axis and tumor microenvironment reveal that gamma-glutamyl hydrolase suppresses glycolysis in colon cancer
Source: Front Immunol. 2022 Dec 7;13:979521. doi: 10.3389/fimmu.2022.979521 (PMC9767965; doi:10.3389/fimmu.2022.979521)
Supplement: Supplementary file 14 [file Table_1.pdf]

Table S1. Genes involved in glycolytic and cholesterogenic pathways were identified from molecular signatures database (mSigDB) and enriched in colon cancer by gene set variation analysis (GSVA).

ID	Gene	Log [FC]	Ave Expr	t	*p.* Value	adj. *p.* Val	B
REACTOME_GLYCOLYSIS (*n* = 69)	AAAS, ADPGK, ALDOA, ALDOB, ALDOC, BPGM, ENO1, ENO2, ENO3, GAPDH, GAPDHS, GCK, GCKR, GNPDA1, GNPDA2, GPI, HK1, HK2, HK3, NDC1, NUP107, NUP133, NUP153, NUP155, NUP160, NUP188, NUP205, NUP210, NUP214, NUP35, NUP37, NUP43, NUP50, NUP54, NUP62, NUP85, NUP88, NUP93, NUP98, PFKFB1, PFKFB2, PFKFB3, PFKFB4, PFKL, PFKM, PFKP, PGAM1, PGK1, PGK2, PGM2L1, PGP, PKLR, PKM, POM121, POM121C, PPP2CA, PPP2CB, PPP2R1A, PPP2R1B, PPP2R5D, PRKACA, PRKACB, PRKACG, RAE1, RANBP2, SEC13, SEH1L, TPI1, TPR	0.495	-0.007	10.623	8.58E-24	1.72E-23	43.148
REACTOME_CHOLESTEROL_ BIOSYNTHESIS (*n* = 24)	ACAT2, ARV1, CYP51A1, DHCR24, DHCR7, EBP, FDFT1, FDPS, GGPS1, HMGCR, HMGCS1, HSD17B7, IDI1, IDI2, LBR, LSS, MSMO1, MVD, MVK, NSDHL, PMVK, SC5D, SQLE, TM7SF2	0.407	-0.014	5.533	5.20E-08	5.20E-08	7.454

Table S2. Protein-protein interaction network was clustered using a Markov cluster algorithm.

Cluster	Cluster color	Gene count	ID	Protein description
Cluster 1	Red	30	AAAS	Aladin; Plays a role in the normal development of the peripheral and central nervous system; Nucleoporins
Cluster 1	Red	30	GCK	Glucokinase; Catalyzes the initial step in utilization of glucose by the beta-cell and liver at physiological glucose concentration. Glucokinase has a high Km for glucose, and so it is effective only when glucose is abundant. The role of GCK is to provide G6P for the synthesis of glycogen. Pancreatic glucokinase plays an important role in modulating insulin secretion. Hepatic glucokinase helps to facilitate the uptake and conversion of glucose by acting as an insulin-sensitive determinant of hepatic glucose usage
Cluster 1	Red	30	GCKR	Glucokinase regulatory protein; Inhibits glucokinase (GCK) by forming an inactive complex with this enzyme. The affinity of GCKR for GCK is modulated by fructose metabolites: GCKR with bound fructose 6- phosphate has increased affinity for GCK, while GCKR with bound fructose 1-phosphate has strongly decreased affinity for GCK and does not inhibit GCK activity
Cluster 1	Red	30	NDC1	Nucleoporin NDC1; Component of the nuclear pore complex (NPC), which plays a key role in de novo assembly and insertion of NPC in the nuclear envelope. Required for NPC and nuclear envelope assembly, possibly by forming a link between the nuclear envelope membrane and soluble nucleoporins, thereby anchoring the NPC in the membrane
Cluster 1	Red	30	NUP107	Nuclear pore complex protein Nup107; Plays a role in the nuclear pore complex (NPC) assembly and/or maintenance. Required for the assembly of peripheral proteins into the NPC. May anchor NUP62 to the NPC; Belongs to the nucleoporin Nup84/Nup107 family
Cluster 1	Red	30	NUP133	Nuclear pore complex protein Nup133; Involved in poly(A)+ RNA transport; Belongs to the nucleoporin Nup133 family
Cluster 1	Red	30	NUP153	Nuclear pore complex protein Nup153; Component of the nuclear pore complex (NPC), a complex required for the trafficking across the nuclear envelope. Functions as a scaffolding element in the nuclear phase of the NPC essential for normal nucleocytoplasmic transport of proteins and mRNAs. Involved in the quality control and retention of unspliced mRNAs in the nucleus; in association with TPR,

				regulates the nuclear export of unspliced mRNA species bearing constitutive transport element (CTE) in a NXF1- and KHDRBS1-independent manner. Mediates TPR anchoring to the nuclear membrane at NPC. [...]
Cluster 1	Red	30	NUP155	Nuclear pore complex protein Nup155; Essential component of nuclear pore complex. Could be essessential for embryogenesis. Nucleoporins may be involved both in binding and translocating proteins during nucleocytoplasmic transport
Cluster 1	Red	30	NUP160	Nuclear pore complex protein Nup160; Involved in poly(A)+ RNA transport; Nucleoporins
Cluster 1	Red	30	NUP188	Nucleoporin NUP188 homolog; May function as a component of the nuclear pore complex (NPC); Armadillo-like helical domain containing
Cluster 1	Red	30	NUP205	Nuclear pore complex protein Nup205; Plays a role in the nuclear pore complex (NPC) assembly and/or maintenance. May anchor NUP62 and other nucleoporins, but not NUP153 and TPR, to the NPC; Armadillo-like helical domain containing
Cluster 1	Red	30	NUP210	Nuclear pore membrane glycoprotein 210; Nucleoporin essential for nuclear pore assembly and fusion, nuclear pore spacing, as well as structural integrity; Nucleoporins
Cluster 1	Red	30	NUP214	Nuclear pore complex protein Nup214; May serve as a docking site in the receptor-mediated import of substrates across the nuclear pore complex; Nucleoporins
Cluster 1	Red	30	NUP35	Nucleoporin NUP53; Functions as a component of the nuclear pore complex (NPC). NPC components, collectively referred to as nucleoporins (NUPs). Can play the role of both NPC structural components and of docking or interaction partners for transiently associated nuclear transport factors. May play a role in the association of MAD1 with the NPC
Cluster 1	Red	30	NUP37	Nucleoporin Nup37; Component of the Nup107-160 subcomplex of the nuclear pore complex (NPC). The Nup107-160 subcomplex is required for the assembly of a functional NPC. The Nup107-160 subcomplex is also required for normal kinetochore microtubule attachment, mitotic progression and chromosome segregation; Nucleoporins
Cluster 1	Red	30	NUP43	Nucleoporin Nup43; Component of the Nup107-160 subcomplex of the nuclear pore complex (NPC). The Nup107-160 subcomplex is required for the assembly of a functional NPC. The Nup107-160

				subcomplex is also required for normal kinetochore microtubule attachment, mitotic progression and chromosome segregation; Nucleoporins
Cluster 1	Red	30	NUP50	Nuclear pore complex protein Nup50; Component of the nuclear pore complex that has a direct role in nuclear protein import. Actively displaces NLSs from importin-alpha, and facilitates disassembly of the importin-alpha:beta-cargo complex and importin recycling. Interacts with regulatory proteins of cell cycle progression including CDKN1B (By similarity). This interaction is required for correct intracellular transport and degradation of CDKN1B (By similarity); Nucleoporins
Cluster 1	Red	30	NUP54	Nucleoporin p54; Component of the nuclear pore complex, a complex required for the trafficking across the nuclear membrane; Nucleoporins
Cluster 1	Red	30	NUP62	Nuclear pore glycoprotein p62; Essential component of the nuclear pore complex. The N-terminal is probably involved in nucleocytoplasmic transport. The C-terminal is involved in protein-protein interaction probably via coiled-coil formation, promotes its association with centrosomes and may function in anchorage of p62 to the pore complex. Plays a role in mitotic cell cycle progression by regulating centrosome segregation, centriole maturation and spindle orientation. It might be involved in protein recruitment to the centrosome after nuclear breakdown; Nucleoporins
Cluster 1	Red	30	NUP85	Nuclear pore complex protein Nup85; Essential component of the nuclear pore complex (NPC) that seems to be required for NPC assembly and maintenance. As part of the NPC Nup107-160 subcomplex plays a role in RNA export and in tethering NUP96/Nup98 and NUP153 to the nucleus. The Nup107-160 complex seems to be required for spindle assembly during mitosis. NUP85 is required for membrane clustering of CCL2-activated CCR2. Seems to be involved in CCR2-mediated chemotaxis of monocytes and may link activated CCR2 to the phosphatidyl-inositol 3-kinase-Rac-lammellipodium protrusion cascade; Nucl [...]
Cluster 1	Red	30	NUP88	Nuclear pore complex protein Nup88; Essential component of nuclear pore complex; Nucleoporins
Cluster 1	Red	30	NUP93	Nuclear pore complex protein Nup93; Plays a role in the nuclear pore complex (NPC) assembly and/or maintenance. May anchor nucleoporins, but not NUP153 and TPR, to the NPC. During renal development, regulates podocyte migration and proliferation through SMAD4 signaling

Cluster 1	Red	30	NUP98	Nuclear pore complex protein Nup98-Nup96; Plays a role in the nuclear pore complex (NPC) assembly and/or maintenance. NUP98 and NUP96 are involved in the bidirectional transport across the NPC. May anchor NUP153 and TPR to the NPC. In cooperation with DHX9, plays a role in transcription and alternative splicing activation of a subset of genes. Involved in the localization of DHX9 in discrete intranuclear foci (GLFG-body); Nucleoporins
Cluster 1	Red	30	POM121	Nuclear envelope pore membrane protein POM 121; Essential component of the nuclear pore complex (NPC). The repeat-containing domain may be involved in anchoring components of the pore complex to the pore membrane. When overexpressed in cells induces the formation of cytoplasmic annulate lamellae (AL); Nucleoporins
Cluster 1	Red	30	POM121C	Nuclear envelope pore membrane protein POM 121C; Essential component of the nuclear pore complex (NPC). The repeat-containing domain may be involved in anchoring components of the pore complex to the pore membrane. When overexpressed in cells induces the formation of cytoplasmic annulate lamellae (AL); Belongs to the POM121 family
Cluster 1	Red	30	RAE1	mRNA export factor; Plays a role in mitotic bipolar spindle formation. Binds mRNA. May function in nucleocytoplasmic transport and in directly or indirectly attaching cytoplasmic mRNPs to the cytoskeleton; Belongs to the WD repeat rae1 family
Cluster 1	Red	30	RANBP2	E3 SUMO-protein ligase RanBP2; E3 SUMO-protein ligase which facilitates SUMO1 and SUMO2 conjugation by UBE2I. Involved in transport factor (Ran-GTP, karyopherin)-mediated protein import via the F-G repeat-containing domain which acts as a docking site for substrates. Binds single- stranded RNA (in vitro). May bind DNA. Component of the nuclear export pathway. Specific docking site for the nuclear export factor exportin-1. Sumoylates PML at 'Lys-490' which is essential for the proper assembly of PML-NB. Recruits BICD2 to the nuclear envelope and cytoplasmic stacks of nuclear pore comple [...]
Cluster 1	Red	30	SEC13	Protein SEC13 homolog; Functions as a component of the nuclear pore complex (NPC) and the COPII coat. At the endoplasmic reticulum, SEC13 is involved in the biogenesis of COPII-coated vesicles; Belongs to the WD repeat SEC13 family

Cluster 1	Red	30	SEH1L	Nucleoporin SEH1; Component of the Nup107-160 subcomplex of the nuclear pore complex (NPC). The Nup107-160 subcomplex is required for the assembly of a functional NPC. The Nup107-160 subcomplex is also required for normal kinetochore microtubule attachment, mitotic progression and chromosome segregation. This subunit plays a role in recruitment of the Nup107-160 subcomplex to the kinetochore; Belongs to the WD repeat SEC13 family
Cluster 1	Red	30	TPR	Nucleoprotein TPR; Component of the nuclear pore complex (NPC), a complex required for the trafficking across the nuclear envelope. Functions as a scaffolding element in the nuclear phase of the NPC essential for normal nucleocytoplasmic transport of proteins and mRNAs, plays a role in the establishment of nuclear-peripheral chromatin compartmentalization in interphase, and in the mitotic spindle checkpoint signaling during mitosis. Involved in the quality control and retention of unspliced mRNAs in the nucleus; in association with NUP153, regulates the nuclear export of unspliced mRNA [...]
Cluster 2	Yellow	29	ADPGK	ADP-dependent glucokinase; Catalyzes the phosphorylation of D-glucose to D-glucose 6-phosphate using ADP as the phosphate donor. GDP and CDP can replace ADP, but with reduced efficiency (By similarity)
Cluster 2	Yellow	29	ALDOA	Fructose-bisphosphate aldolase A; Plays a key role in glycolysis and gluconeogenesis. In addition, may also function as scaffolding protein (By similarity); Belongs to the class I fructose-bisphosphate aldolase family
Cluster 2	Yellow	29	ALDOB	Belongs to the class I fructose-bisphosphate aldolase family
Cluster 2	Yellow	29	ALDOC	Belongs to the class I fructose-bisphosphate aldolase family
Cluster 2	Yellow	29	BPGM	Bisphosphoglycerate mutase; Plays a major role in regulating hemoglobin oxygen affinity by controlling the levels of its allosteric effector 2,3- bisphosphoglycerate (2,3-BPG). Also exhibits mutase (EC 5.4.2.1) activity; Belongs to the phosphoglycerate mutase family. BPG- dependent PGAM subfamily
Cluster 2	Yellow	29	ENO1	Alpha-enolase; Multifunctional enzyme that, as well as its role in glycolysis, plays a part in various processes such as growth control, hypoxia tolerance and allergic responses. May also function in the intravascular and pericellular fibrinolytic system due to its ability to serve as a receptor and activator of

				plasminogen on the cell surface of several cell-types such as leukocytes and neurons. Stimulates immunoglobulin production; Belongs to the enolase family
Cluster 2	Yellow	29	ENO2	Gamma-enolase; Has neurotrophic and neuroprotective properties on a broad spectrum of central nervous system (CNS) neurons. Binds, in a calcium-dependent manner, to cultured neocortical neurons and promotes cell survival (By similarity); Enolases
Cluster 2	Yellow	29	ENO3	Beta-enolase; Appears to have a function in striated muscle development and regeneration; Belongs to the enolase family
Cluster 2	Yellow	29	GAPDH	Glyceraldehyde-3-phosphate dehydrogenase; Has both glyceraldehyde-3-phosphate dehydrogenase and nitrosylase activities, thereby playing a role in glycolysis and nuclear functions, respectively. Participates in nuclear events including transcription, RNA transport, DNA replication and apoptosis. Nuclear functions are probably due to the nitrosylase activity that mediates cysteine S-nitrosylation of nuclear target proteins such as SIRT1, HDAC2 and PRKDC. Modulates the organization and assembly of the cytoskeleton. Facilitates the CHP1-dependent microtubule and membrane associations throu [...]
Cluster 2	Yellow	29	GAPDHS	Glyceraldehyde-3-phosphate dehydrogenase, testis-specific; May play an important role in regulating the switch between different pathways for energy production during spermiogenesis and in the spermatozoon. Required for sperm motility and male fertility (By similarity)
Cluster 2	Yellow	29	GNPDA1	Glucosamine-6-phosphate isomerase 1; Seems to trigger calcium oscillations in mammalian eggs. These oscillations serve as the essential trigger for egg activation and early development of the embryo (By similarity); Belongs to the glucosamine/galactosamine-6-phosphate isomerase family
Cluster 2	Yellow	29	GNPDA2	Glucosamine-6-phosphate deaminase 2
Cluster 2	Yellow	29	GPI	Glucose-6-phosphate isomerase; Besides it's role as a glycolytic enzyme, mammalian GPI can function as a tumor-secreted cytokine and an angiogenic factor (AMF) that stimulates endothelial cell motility. GPI is also a neurotrophic factor (Neuroleukin) for spinal and sensory neurons
Cluster 2	Yellow	29	HK1	Catalyzes the phosphorylation of various hexoses, such as D- glucose, D-glucosamine, D-fructose, D-mannose and 2-deoxy-D-glucose, to hexose 6-phosphate (D-glucose 6-phosphate, D-glucosamine 6-phosphate, D-fructose 6-phosphate, D-mannose 6-phosphate and 2-deoxy-D-glucose 6- phosphate,

				respectively) (PubMed:1637300, PubMed:25316723, PubMed:27374331). Does not phosphorylate N-acetyl-D-glucosamine. Mediates the initial step of glycolysis by catalyzing phosphorylation of D-glucose to D-glucose 6-phosphate (By similarity). Involved in innate immunity and inflammation by acting as a pattern [...]
Cluster 2	Yellow	29	HK2	Hexokinase-2; Hexokinase 2; Belongs to the hexokinase family
Cluster 2	Yellow	29	HK3	Catalyzes the phosphorylation of hexose, such as D-glucose and D-fructose, to hexose 6-phosphate (D-glucose 6-phosphate and D- fructose 6-phosphate, respectively). Mediates the initial step of glycolysis by catalyzing phosphorylation of D-glucose to D-glucose 6-phosphate.
Cluster 2	Yellow	29	PFKFB3	6-phosphofructo-2-kinase/fructose-2,6-bisphosphatase 3; Synthesis and degradation of fructose 2,6-bisphosphate; In the C-terminal section; belongs to the phosphoglycerate mutase family
Cluster 2	Yellow	29	PFKFB4	6-phosphofructo-2-kinase/fructose-2,6-bisphosphatase 4; Synthesis and degradation of fructose 2,6-bisphosphate; In the C-terminal section; belongs to the phosphoglycerate mutase family
Cluster 2	Yellow	29	PFKL	ATP-dependent 6-phosphofructokinase, liver type; Catalyzes the phosphorylation of D-fructose 6-phosphate to fructose 1,6-bisphosphate by ATP, the first committing step of glycolysis; Belongs to the phosphofructokinase type A (PFKA) family. ATP-dependent PFK group I subfamily. Eukaryotic two domain clade "E" sub-subfamily
Cluster 2	Yellow	29	PFKM	ATP-dependent 6-phosphofructokinase, muscle type; Catalyzes the phosphorylation of D-fructose 6-phosphate to fructose 1,6-bisphosphate by ATP, the first committing step of glycolysis; Belongs to the phosphofructokinase type A (PFKA) family. ATP-dependent PFK group I subfamily. Eukaryotic two domain clade "E" sub-subfamily
Cluster 2	Yellow	29	PFKP	ATP-dependent 6-phosphofructokinase, platelet type; Catalyzes the phosphorylation of D-fructose 6-phosphate to fructose 1,6-bisphosphate by ATP, the first committing step of glycolysis; Belongs to the phosphofructokinase type A (PFKA) family. ATP-dependent PFK group I subfamily. Eukaryotic two domain clade "E" sub-subfamily

Cluster 2	Yellow	29	PGAM1	Phosphoglycerate mutase 1; Interconversion of 3- and 2-phosphoglycerate with 2,3-bisphosphoglycerate as the primer of the reaction. Can also catalyze the reaction of EC 5.4.2.4 (synthase), but with a reduced activity
Cluster 2	Yellow	29	PGK1	Phosphoglycerate kinase 1; In addition to its role as a glycolytic enzyme, it seems that PGK-1 acts as a polymerase alpha cofactor protein (primer recognition protein). May play a role in sperm motility
Cluster 2	Yellow	29	PGK2	Phosphoglycerate kinase 2; Essential for sperm motility and male fertility. Not required for the completion of spermatogenesis (By similarity); Belongs to the phosphoglycerate kinase family
Cluster 2	Yellow	29	PGM2L1	Glucose 1,6-bisphosphate synthase; Glucose 1,6-bisphosphate synthase using 1,3- bisphosphoglycerate as a phosphate donor and a series of 1- phosphate sugars as acceptors, including glucose 1-phosphate, mannose 1-phosphate, ribose 1-phosphate and deoxyribose 1- phosphate. 5 or 6-phosphosugars are bad substrates, with the exception of glucose 6-phosphate. Also synthesizes ribose 1,5- bisphosphate. Has only low phosphopentomutase and phosphoglucomutase activities
Cluster 2	Yellow	29	PGP	Glycerol-3-phosphate phosphatase; Glycerol-3-phosphate phosphatase hydrolyzing glycerol-3-phosphate into glycerol. Thereby, regulates the cellular levels of glycerol-3-phosphate a metabolic intermediate of glucose, lipid and energy metabolism. Was also shown to have a 2-phosphoglycolate phosphatase activity and a tyrosine-protein phosphatase activity. However, their physiological relevance is unclear. In vitro, has also a phosphatase activity toward ADP, ATP, GDP and GTP (By similarity)
Cluster 2	Yellow	29	PKLR	Pyruvate kinase PKLR; Plays a key role in glycolysis; Belongs to the pyruvate kinase family
Cluster 2	Yellow	29	PKM	Pyruvate kinase PKM; Glycolytic enzyme that catalyzes the transfer of a phosphoryl group from phosphoenolpyruvate (PEP) to ADP, generating ATP. Stimulates POU5F1-mediated transcriptional activation. Plays a general role in caspase independent cell death of tumor cells. The ratio between the highly active tetrameric form and nearly inactive dimeric form determines whether glucose carbons are channeled to biosynthetic processes or used for glycolytic ATP production. The transition between the 2 forms contributes to the control of glycolysis and is important for tumor cell proliferation a [...]
Cluster 2	Yellow	29	TPI1	Triosephosphate isomerase 1; Belongs to the triosephosphate isomerase family
Cluster 3	Green	23	ACAT2	Acetyl-CoA acetyltransferase, cytosolic; acetyl-CoA acetyltransferase 2

Cluster 3	Green	23	CYP51A1	Lanosterol 14-alpha demethylase; Catalyzes C14-demethylation of lanosterol; it transforms lanosterol into 4,4'-dimethyl cholesta-8,14,24-triene-3-beta-ol; Cytochrome P450 family 51
Cluster 3	Green	23	DHCR24	Delta (24)-sterol reductase; Catalyzes the reduction of the delta-24 double bond of sterol intermediates. Protects cells from oxidative stress by reducing caspase 3 activity during apoptosis induced by oxidative stress. Also protects against amyloid-beta peptide-induced apoptosis; Belongs to the FAD-binding oxidoreductase/transferase type 4 family
Cluster 3	Green	23	DHCR7	7-dehydrocholesterol reductase; Production of cholesterol by reduction of C7-C8 double bond of 7-dehydrocholesterol (7-DHC); Belongs to the ERG4/ERG24 family
Cluster 3	Green	23	EBP	3-beta-hydroxysteroid-Delta(8), Delta(7)-isomerase; Catalyzes the conversion of Delta(8)-sterols to their corresponding Delta(7)-isomers; Belongs to the EBP family
Cluster 3	Green	23	FDFT1	Squalene synthase; Farnesyl-diphosphate farnesyltransferase 1
Cluster 3	Green	23	FDPS	Farnesyl pyrophosphate synthase; Key enzyme in isoprenoid biosynthesis which catalyzes the formation of farnesyl diphosphate (FPP), a precursor for several classes of essential metabolites including sterols, dolichols, carotenoids, and ubiquinones. FPP also serves as substrate for protein farnesylation and geranylgeranylation. Catalyzes the sequential condensation of isopentenyl pyrophosphate with the allylic pyrophosphates, dimethylallyl pyrophosphate, and then with the resultant geranylpyrophosphate to the ultimate product farnesyl pyrophosphate
Cluster 3	Green	23	GGPS1	Geranylgeranyl pyrophosphate synthase; Catalyzes the trans-addition of the three molecules of IPP onto DMAPP to form geranylgeranyl pyrophosphate, an important precursor of carotenoids and geranylated proteins
Cluster 3	Green	23	HMGCR	3-hydroxy-3-methylglutaryl-coenzyme A reductase; Transmembrane glycoprotein that is the rate-limiting enzyme in cholesterol biosynthesis as well as in the biosynthesis of nonsterol isoprenoids that are essential for normal cell function including ubiquinone and geranylgeranyl proteins; Belongs to the HMG-CoA reductase family
Cluster 3	Green	23	HMGCS1	Hydroxymethylglutaryl-CoA synthase, cytoplasmic; This enzyme condenses acetyl-CoA with acetoacetyl-CoA to form HMG-CoA, which is the substrate for HMG-CoA reductase

Cluster 3	Green	23	HSD17B7	3-keto-steroid reductase; Responsible for the reduction of the keto group on the C-3 of sterols; Short chain dehydrogenase/reductase superfamily
Cluster 3	Green	23	IDI1	Isopentenyl-diphosphate Delta-isomerase 1; Catalyzes the 1,3-allylic rearrangement of the homoallylic substrate isopentenyl (IPP) to its highly electrophilic allylic isomer, dimethylallyl diphosphate (DMAPP)
Cluster 3	Green	23	IDI2	Isopentenyl-diphosphate delta-isomerase 2; Catalyzes the 1,3-allylic rearrangement of the homoallylic substrate isopentenyl (IPP) to its highly electrophilic allylic isomer, dimethylallyl diphosphate (DMAPP)
Cluster 3	Green	23	LBR	Lamin-B receptor; Anchors the lamina and the heterochromatin to the inner nuclear membrane; Tudor domain containing
Cluster 3	Green	23	LSS	Lanosterol synthase; Catalyzes the cyclization of (S)-2,3 oxidosqualene to lanosterol, a reaction that forms the sterol nucleus. Through the production of lanosterol may regulate lens protein aggregation and increase transparency; Belongs to the terpene cyclase/mutase family
Cluster 3	Green	23	MSMO1	Methylsterol monooxygenase 1; Catalyzes the first step in the removal of the two C-4 methyl groups of 4,4-dimethylzymosterol; Belongs to the sterol desaturase family
Cluster 3	Green	23	MVD	Diphosphomevalonate decarboxylase; Performs the first committed step in the biosynthesis of isoprenes
Cluster 3	Green	23	MVK	Mevalonate kinase; May be a regulatory site in cholesterol biosynthetic pathway; Belongs to the GHMP kinase family. Mevalonate kinase subfamily
Cluster 3	Green	23	NSDHL	Sterol-4-alpha-carboxylate 3-dehydrogenase, decarboxylating; Involved in the sequential removal of two C-4 methyl groups in post-squalene cholesterol biosynthesis; Short chain dehydrogenase/reductase superfamily
Cluster 3	Green	23	PMVK	Phosphomevalonate kinase
Cluster 3	Green	23	SC5D	Lathosterol oxidase; Catalyzes a dehydrogenation to introduce C5-6 double bond into lathosterol; Belongs to the sterol desaturase family
Cluster 3	Green	23	SQLE	Squalene monooxygenase; Catalyzes the first oxygenation step in sterol biosynthesis and is suggested to be one of the rate-limiting enzymes in this pathway; Belongs to the squalene monooxygenase family

Cluster 3	Green	23	TM7SF2	Delta (14)-sterol reductase; Involved in the conversion of lanosterol to cholesterol
Cluster 4	Blue	10	PFKFB1	6-phosphofructo-2-kinase/fructose-2,6-bisphosphatase 1; Synthesis and degradation of fructose 2,6-bisphosphate; In the C-terminal section; belongs to the phosphoglycerate mutase family
Cluster 4	Blue	10	PFKFB2	6-phosphofructo-2-kinase/fructose-2,6-bisphosphatase 2; Synthesis and degradation of fructose 2,6-bisphosphate; In the C-terminal section; belongs to the phosphoglycerate mutase family
Cluster 4	Blue	10	PPP2CA	Serine/threonine-protein phosphatase 2A catalytic subunit alpha isoform; PP2A is the major phosphatase for microtubule-associated proteins (MAPs). PP2A can modulate the activity of phosphorylase B kinase casein kinase 2, mitogen-stimulated S6 kinase, and MAP-2 kinase. Cooperates with SGO2 to protect centromeric cohesin from separase-mediated cleavage in oocytes specifically during meiosis I (By similarity). Can dephosphorylate SV40 large T antigen and p53/TP53. Activates RAF1 by dephosphorylating it at 'Ser-259'; Belongs to the PPP phosphatase family. PP-1 subfamily
Cluster 4	Blue	10	PPP2CB	Serine/threonine-protein phosphatase 2A catalytic subunit beta isoform; PP2A can modulate the activity of phosphorylase B kinase casein kinase 2, mitogen-stimulated S6 kinase, and MAP-2 kinase; Protein phosphatase catalytic subunits
Cluster 4	Blue	10	PPP2R1A	Serine/threonine-protein phosphatase 2A 65 kDa regulatory subunit A alpha isoform; The PR65 subunit of protein phosphatase 2A serves as a scaffolding molecule to coordinate the assembly of the catalytic subunit and a variable regulatory B subunit. Upon interaction with GNA12 promotes dephosphorylation of microtubule associated protein TAU/MAPT. Required for proper chromosome segregation and for centromeric localization of SGO1 in mitosis
Cluster 4	Blue	10	PPP2R1B	Serine/threonine-protein phosphatase 2A 65 kDa regulatory subunit A beta isoform; The PR65 subunit of protein phosphatase 2A serves as a scaffolding molecule to coordinate the assembly of the catalytic subunit and a variable regulatory B subunit; Armadillo-like helical domain containing
Cluster 4	Blue	10	PPP2R5D	Serine/threonine-protein phosphatase 2A 56 kDa regulatory subunit delta isoform; The B regulatory subunit might modulate substrate selectivity and catalytic activity, and also might direct the localization of the catalytic enzyme to a particular subcellular compartment; Belongs to the phosphatase 2A regulatory subunit B56 family

Cluster 4	Blue	10	PRKACA	cAMP-dependent protein kinase catalytic subunit alpha; Phosphorylates a large number of substrates in the cytoplasm and the nucleus. Regulates the abundance of compartmentalized pools of its regulatory subunits through phosphorylation of PJA2 which binds and ubiquitinates these subunits, leading to their subsequent proteolysis. Phosphorylates CDC25B, ABL1, NFKB1, CLDN3, PSMC5/RPT6, PJA2, RYR2, RORA and VASP. RORA is activated by phosphorylation. Required for glucose-mediated adipogenic differentiation increase and osteogenic differentiation inhibition from osteoblasts.
Cluster 4	Blue	10	PRKACB	cAMP-dependent protein kinase catalytic subunit beta; Mediates cAMP-dependent signaling triggered by receptor binding to GPCRs. PKA activation regulates diverse cellular processes such as cell proliferation, the cell cycle, differentiation and regulation of microtubule dynamics, chromatin condensation and decondensation, nuclear envelope disassembly and reassembly, as well as regulation of intracellular transport mechanisms and ion flux. Regulates the abundance of compartmentalized pools of its regulatory subunits through phosphorylation of PJA2.
Cluster 4	Blue	10	PRKACG	cAMP-dependent protein kinase catalytic subunit gamma; Phosphorylates a large number of substrates in the cytoplasm and the nucleus; Belongs to the protein kinase superfamily. AGC Ser/Thr protein kinase family. cAMP subfamily

Table S3. Detailed list of survival outcomes of cases obtained from TCGA-COAD.

ID	Cluster	Subtype	OS	OS.time	DSS	DSS.time	DFI	DFI.time	PFI	PFI.time
TCGA-AA-3975-01	Cluster 1	Quiescent	0	2.84	0	2.84	0	2.84	0	2.84
TCGA-AA-3680-01	Cluster 1	Quiescent	1	0.92	1	0.92	NA	NA	1	0.92
TCGA-G4-6310-01	Cluster 1	Quiescent	0	5.30	0	5.30	0	5.30	0	5.30
TCGA-A6-6781-01	Cluster 1	Quiescent	0	1.64	0	1.64	0	1.64	0	1.64
TCGA-A6-5657-01	Cluster 1	Quiescent	0	2.64	0	2.64	0	2.64	0	2.64
TCGA-CM-5344-01	Cluster 1	Quiescent	0	1.84	0	1.84	NA	NA	0	1.84
TCGA-F4-6854-01	Cluster 1	Quiescent	0	0.04	0	0.04	0	0.04	0	0.04
TCGA-D5-6922-01	Cluster 1	Quiescent	0	0.84	0	0.84	NA	NA	0	0.84
TCGA-DM-A1D9-01	Cluster 1	Quiescent	0	11.70	0	11.70	NA	NA	0	11.70
TCGA-F4-6703-01	Cluster 1	Quiescent	0	3.99	0	3.99	0	3.99	0	3.99
TCGA-A6-A567-01	Cluster 1	Quiescent	1	5.15	1	5.15	NA	NA	1	1.68
TCGA-D5-6924-01	Cluster 1	Quiescent	0	1.19	0	1.19	NA	NA	0	1.19
TCGA-AA-A004-01	Cluster 1	Quiescent	0	1.16	0	1.16	0	1.16	0	1.16
TCGA-G4-6320-01	Cluster 1	Quiescent	0	2.20	0	2.20	NA	NA	0	2.20
TCGA-A6-6654-01	Cluster 1	Quiescent	0	1.99	0	1.99	0	1.99	0	1.99
TCGA-AA-3833-01	Cluster 1	Quiescent	0	1.33	0	1.33	0	1.33	0	1.33
TCGA-A6-6652-01	Cluster 1	Quiescent	0	2.06	0	2.06	NA	NA	0	2.06
TCGA-F4-6806-01	Cluster 1	Quiescent	0	3.45	0	3.45	1	0.92	1	0.92
TCGA-CM-6680-01	Cluster 1	Quiescent	0	1.00	0	1.00	NA	NA	0	1.00
TCGA-A6-A566-01	Cluster 1	Quiescent	1	2.08	1	2.08	NA	NA	1	0.70
TCGA-F4-6570-01	Cluster 1	Quiescent	1	0.52	0	0.52	NA	NA	0	0.52
TCGA-CM-6161-01	Cluster 1	Quiescent	0	1.25	0	1.25	NA	NA	0	1.25
TCGA-CK-6748-01	Cluster 1	Quiescent	0	0.17	0	0.17	NA	NA	0	0.17
TCGA-F4-6809-01	Cluster 1	Quiescent	1	1.10	1	1.10	NA	NA	1	1.10

TCGA-AZ-5407-01	Cluster 1	Quiescent	0	7.35	0	7.35	0	7.35	0	7.35
TCGA-CM-6170-01	Cluster 1	Quiescent	0	1.25	0	1.25	NA	NA	0	1.25
TCGA-AA-A02H-01	Cluster 1	Quiescent	1	0.17	1	0.17	NA	NA	1	0.17
TCGA-G4-6294-01	Cluster 1	Quiescent	1	2.35	1	2.35	NA	NA	1	1.42
TCGA-D5-6530-01	Cluster 1	Quiescent	0	1.70	0	1.70	NA	NA	0	1.70
TCGA-AA-3972-01	Cluster 1	Quiescent	0	4.25	0	4.25	NA	NA	1	3.33
TCGA-D5-6534-01	Cluster 1	Quiescent	0	3.61	0	3.61	0	3.61	0	3.61
TCGA-WS-AB45-01	Cluster 1	Quiescent	0	5.84	0	5.84	0	5.84	0	5.84
TCGA-A6-6649-01	Cluster 1	Quiescent	0	2.01	0	2.01	0	2.01	0	2.01
TCGA-AD-6899-01	Cluster 1	Quiescent	1	0.48	0	0.48	0	0.48	0	0.48
TCGA-AA-3511-01	Cluster 1	Quiescent	0	0.58	0	0.58	1	0.16	1	0.16
TCGA-AZ-6605-01	Cluster 1	Quiescent	1	0.44	0	0.44	NA	NA	0	0.44
TCGA-NH-A50U-01	Cluster 1	Quiescent	1	0.92	1	0.92	NA	NA	1	0.76
TCGA-AA-3530-01	Cluster 1	Quiescent	0	1.59	0	1.59	0	1.59	0	1.59
TCGA-CM-6167-01	Cluster 1	Quiescent	0	1.25	0	1.25	NA	NA	0	1.25
TCGA-CM-5864-01	Cluster 1	Quiescent	0	1.25	0	1.25	NA	NA	0	1.25
TCGA-AD-A5EK-01	Cluster 1	Quiescent	0	1.37	0	1.37	0	1.37	0	1.37
TCGA-AZ-6599-01	Cluster 1	Quiescent	1	0.56	0	0.56	0	0.56	0	0.56
TCGA-CM-5348-01	Cluster 1	Quiescent	0	1.92	0	1.92	NA	NA	0	1.92
TCGA-AA-3973-01	Cluster 1	Quiescent	0	1.09	0	1.09	NA	NA	0	1.09
TCGA-AY-4071-01	Cluster 1	Quiescent	1	0.08	0	0.08	NA	NA	0	0.08
TCGA-AA-3870-01	Cluster 1	Quiescent	0	2.50	0	2.50	NA	NA	0	2.50
TCGA-NH-A6GC-01	Cluster 1	Quiescent	0	1.07	0	1.07	NA	NA	0	1.07
TCGA-F4-6807-01	Cluster 1	Quiescent	0	3.59	0	3.59	0	3.59	0	3.59
TCGA-AA-3971-01	Cluster 1	Quiescent	0	1.34	0	1.34	0	1.34	0	1.34
TCGA-AA-A017-01	Cluster 1	Quiescent	0	1.25	0	1.25	0	1.25	0	1.25

TCGA-AA-3952-01	Cluster 1	Quiescent	1	0.17	1	0.17	NA	NA	1	0.17
TCGA-A6-6651-01	Cluster 1	Quiescent	0	1.81	0	1.81	NA	NA	0	1.81
TCGA-3L-AA1B-01	Cluster 1	Quiescent	0	1.30	0	1.30	0	1.30	0	1.30
TCGA-AA-3696-01	Cluster 1	Quiescent	1	0.42	1	0.42	NA	NA	1	0.42
TCGA-AZ-4323-01	Cluster 1	Quiescent	1	0.12	1	0.12	NA	NA	1	0.12
TCGA-NH-A50V-01	Cluster 1	Quiescent	0	1.61	0	1.61	0	1.61	0	1.61
TCGA-A6-2686-01	Cluster 1	Quiescent	1	3.08	0	3.08	0	3.08	0	3.08
TCGA-G4-6297-01	Cluster 1	Quiescent	0	6.87	0	6.87	NA	NA	1	1.20
TCGA-AA-3862-01	Cluster 1	Quiescent	0	2.50	0	2.50	0	2.50	0	2.50
TCGA-A6-5664-01	Cluster 1	Quiescent	0	1.84	0	1.84	NA	NA	1	0.50
TCGA-D5-6538-01	Cluster 1	Quiescent	0	1.43	0	1.43	NA	NA	0	1.43
TCGA-G4-6625-01	Cluster 1	Quiescent	0	7.65	0	7.65	NA	NA	1	7.02
TCGA-CM-5862-01	Cluster 1	Quiescent	1	0.42	1	0.42	NA	NA	1	0.08
TCGA-A6-2685-01	Cluster 1	Quiescent	0	3.10	0	3.10	NA	NA	1	2.60
TCGA-5M-AATE-01	Cluster 1	Quiescent	0	3.29	0	3.29	NA	NA	1	2.22
TCGA-AU-3779-01	Cluster 1	Quiescent	0	1.21	0	1.21	NA	NA	0	1.21
TCGA-F4-6463-01	Cluster 1	Quiescent	0	2.98	0	2.98	0	2.98	0	2.98
TCGA-AA-3692-01	Cluster 1	Quiescent	1	3.00	1	3.00	NA	NA	1	1.08
TCGA-AA-A03J-01	Cluster 1	Quiescent	0	3.41	0	3.41	0	3.41	0	3.41
TCGA-AA-3494-01	Cluster 1	Quiescent	0	0.08	0	0.08	NA	NA	0	0.08
TCGA-AD-6964-01	Cluster 1	Quiescent	1	0.91	1	0.91	NA	NA	1	0.89
TCGA-A6-3810-01	Cluster 1	Quiescent	0	3.04	0	3.04	0	3.04	0	3.04
TCGA-AZ-6607-01	Cluster 1	Quiescent	1	0.27	1	0.27	NA	NA	1	0.27
TCGA-A6-2674-01	Cluster 1	Quiescent	0	3.65	0	3.65	NA	NA	1	2.01
TCGA-A6-6142-01	Cluster 1	Quiescent	0	2.09	0	2.09	NA	NA	1	1.79
TCGA-F4-6460-01	Cluster 1	Quiescent	1	2.66	1	2.66	1	0.86	1	0.86

TCGA-DM-A1D8-01	Cluster 1	Quiescent	1	1.05	NA	1.05	NA	NA	0	1.05
TCGA-NH-A6GA-01	Cluster 1	Quiescent	1	0.83	1	0.83	NA	NA	1	0.02
TCGA-AA-A01D-01	Cluster 1	Quiescent	1	0.92	1	0.92	NA	NA	1	0.50
TCGA-AY-6196-01	Cluster 1	Quiescent	0	0.02	0	0.02	NA	NA	0	0.02
TCGA-F4-6704-01	Cluster 1	Quiescent	0	0.13	0	0.13	NA	NA	0	0.13
TCGA-CM-6165-01	Cluster 1	Quiescent	0	1.34	0	1.34	NA	NA	0	1.34
TCGA-G4-6323-01	Cluster 1	Quiescent	0	1.15	0	1.15	NA	NA	0	1.15
TCGA-A6-5656-01	Cluster 1	Quiescent	0	2.74	0	2.74	0	2.74	0	2.74
TCGA-AA-3812-01	Cluster 1	Quiescent	0	2.92	0	2.92	1	2.09	1	2.09
TCGA-AA-3662-01	Cluster 1	Quiescent	0	0.50	0	0.50	NA	NA	0	0.50
TCGA-AD-6901-01	Cluster 1	Quiescent	1	1.87	1	1.87	1	1.28	1	1.28
TCGA-D5-6541-01	Cluster 1	Quiescent	0	1.30	0	1.30	NA	NA	0	1.30
TCGA-A6-A565-01	Cluster 1	Quiescent	1	1.35	1	1.35	NA	NA	1	0.82
TCGA-NH-A8F7-01	Cluster 1	Quiescent	0	1.49	0	1.49	NA	NA	0	1.49
TCGA-AA-A00D-01	Cluster 1	Quiescent	0	1.58	0	1.58	0	1.58	0	1.58
TCGA-F4-6855-01	Cluster 1	Quiescent	0	3.95	0	3.95	0	3.95	0	3.95
TCGA-G4-6627-01	Cluster 1	Quiescent	0	6.23	0	6.23	1	2.75	1	2.75
TCGA-F4-6461-01	Cluster 1	Quiescent	1	0.93	1	0.93	NA	NA	1	0.79
TCGA-D5-6928-01	Cluster 1	Quiescent	0	0.97	0	0.97	NA	NA	0	0.97
TCGA-AA-A00F-01	Cluster 1	Quiescent	0	2.84	0	2.84	NA	NA	0	2.84
TCGA-5M-AAT6-01	Cluster 1	Quiescent	1	0.79	1	0.79	NA	NA	1	0.60
TCGA-DM-A28E-01	Cluster 1	Quiescent	0	9.99	0	9.99	NA	NA	0	9.99
TCGA-D5-5539-01	Cluster 1	Quiescent	0	1.63	0	1.63	NA	NA	1	1.59
TCGA-AA-A00O-01	Cluster 1	Quiescent	0	2.25	0	2.25	NA	NA	0	2.25
TCGA-AA-A00L-01	Cluster 1	Quiescent	0	3.17	0	3.17	0	3.17	0	3.17
TCGA-AA-A01P-01	Cluster 1	Quiescent	1	3.17	1	3.17	NA	NA	1	2.09

TCGA-CM-6679-01	Cluster 1	Quiescent	0	0.84	0	0.84	NA	NA	0	0.84
TCGA-AY-A71X-01	Cluster 1	Quiescent	0	1.61	0	1.61	0	1.61	0	1.61
TCGA-AA-3489-01	Cluster 1	Quiescent	1	0.59	1	0.59	NA	NA	1	0.59
TCGA-G4-6293-01	Cluster 1	Quiescent	0	11.10	0	11.10	0	11.10	0	11.10
TCGA-AA-3517-01	Cluster 1	Quiescent	0	3.25	0	3.25	1	2.25	1	2.25
TCGA-D5-6536-01	Cluster 1	Quiescent	0	1.49	0	1.49	NA	NA	1	1.35
TCGA-CM-4743-01	Cluster 1	Quiescent	0	1.92	0	1.92	0	1.92	0	1.92
TCGA-AA-A02W-01	Cluster 1	Quiescent	0	3.42	0	3.42	1	2.75	1	2.75
TCGA-CM-4752-01	Cluster 1	Quiescent	0	1.08	0	1.08	0	1.08	0	1.08
TCGA-AA-3552-01	Cluster 1	Quiescent	1	1.08	0	1.08	1	0.33	1	0.33
TCGA-CM-5863-01	Cluster 1	Quiescent	0	1.25	0	1.25	NA	NA	0	1.25
TCGA-AA-3522-01	Cluster 1	Quiescent	0	3.09	0	3.09	0	3.09	0	3.09
TCGA-A6-2675-01	Cluster 1	Quiescent	0	3.62	0	3.62	0	3.62	0	3.62
TCGA-AA-3496-01	Cluster 1	Quiescent	0	0.08	0	0.08	NA	NA	0	0.08
TCGA-CM-6169-01	Cluster 1	Quiescent	0	1.08	0	1.08	NA	NA	0	1.08
TCGA-CM-6164-01	Cluster 1	Quiescent	0	2.42	0	2.42	NA	NA	0	2.42
TCGA-D5-6926-01	Cluster 1	Quiescent	0	0.75	0	0.75	NA	NA	0	0.75
TCGA-AA-A00R-01	Cluster 2	Glycolytic	0	0.08	0	0.08	NA	NA	0	0.08
TCGA-G4-6304-01	Cluster 2	Glycolytic	0	4.47	0	4.47	NA	NA	1	2.35
TCGA-AA-3710-01	Cluster 2	Glycolytic	0	2.25	0	2.25	0	2.25	0	2.25
TCGA-AA-3854-01	Cluster 2	Glycolytic	0	3.00	0	3.00	0	3.00	0	3.00
TCGA-CK-5916-01	Cluster 2	Glycolytic	1	1.76	1	1.76	NA	NA	1	0.80
TCGA-AA-3818-01	Cluster 2	Glycolytic	1	0.08	1	0.08	NA	NA	1	0.08
TCGA-AA-3549-01	Cluster 2	Glycolytic	0	1.75	0	1.75	0	1.75	0	1.75
TCGA-CA-5254-01	Cluster 2	Glycolytic	0	1.06	0	1.06	0	1.06	0	1.06
TCGA-A6-5665-01	Cluster 2	Glycolytic	0	1.84	0	1.84	NA	NA	1	1.45

TCGA-AA-3875-01	Cluster 2	Glycolytic	0	1.50	0	1.50	NA	NA	0	1.50
TCGA-AA-A02R-01	Cluster 2	Glycolytic	1	1.84	1	1.84	NA	NA	1	1.84
TCGA-AA-A02O-01	Cluster 2	Glycolytic	0	0.08	0	0.08	NA	NA	0	0.08
TCGA-AM-5821-01	Cluster 2	Glycolytic	0	0.08	0	0.08	NA	NA	0	0.08
TCGA-AA-A00Q-01	Cluster 2	Glycolytic	0	3.50	0	3.50	0	3.50	0	3.50
TCGA-CM-6162-01	Cluster 2	Glycolytic	0	1.00	0	1.00	NA	NA	0	1.00
TCGA-CM-5349-01	Cluster 2	Glycolytic	0	2.51	0	2.51	NA	NA	0	2.51
TCGA-CA-6719-01	Cluster 2	Glycolytic	0	1.19	0	1.19	NA	NA	1	0.97
TCGA-G4-6299-01	Cluster 2	Glycolytic	0	6.21	0	6.21	NA	NA	0	6.21
TCGA-DM-A1DB-01	Cluster 2	Glycolytic	1	3.69	NA	3.69	NA	NA	0	3.69
TCGA-A6-A56B-01	Cluster 2	Glycolytic	1	4.69	0	4.69	1	4.60	1	4.60
TCGA-D5-6537-01	Cluster 2	Glycolytic	1	0.40	1	0.40	NA	NA	1	0.34
TCGA-G4-6586-01	Cluster 2	Glycolytic	0	2.98	0	2.98	NA	NA	0	2.98
TCGA-AA-A00J-01	Cluster 2	Glycolytic	0	1.50	0	1.50	NA	NA	0	1.50
TCGA-AZ-4616-01	Cluster 2	Glycolytic	1	0.43	1	0.43	NA	NA	1	0.43
TCGA-AU-6004-01	Cluster 2	Glycolytic	0	2.26	0	2.26	NA	NA	0	2.26
TCGA-AA-3506-01	Cluster 2	Glycolytic	0	4.84	0	4.84	NA	NA	0	4.84
TCGA-AA-3715-01	Cluster 2	Glycolytic	1	1.59	1	1.59	NA	NA	1	0.42
TCGA-AA-3966-01	Cluster 2	Glycolytic	0	0.17	0	0.17	NA	NA	0	0.17
TCGA-DM-A1HA-01	Cluster 2	Glycolytic	0	10.96	0	10.96	NA	NA	0	10.96
TCGA-AA-3502-01	Cluster 2	Glycolytic	0	2.92	0	2.92	0	2.92	0	2.92
TCGA-AA-A02F-01	Cluster 2	Glycolytic	0	3.33	0	3.33	NA	NA	0	3.33
TCGA-CM-6171-01	Cluster 2	Glycolytic	0	1.17	0	1.17	NA	NA	0	1.17
TCGA-AA-3518-01	Cluster 2	Glycolytic	0	0.08	0	0.08	NA	NA	0	0.08
TCGA-DM-A28M-01	Cluster 2	Glycolytic	0	7.93	0	7.93	NA	NA	0	7.93
TCGA-F4-6459-01	Cluster 2	Glycolytic	1	0.72	0	0.72	NA	NA	0	0.72

TCGA-AA-3968-01	Cluster 2	Glycolytic	0	1.83	0	1.83	0	1.83	0	1.83
TCGA-AA-3947-01	Cluster 2	Glycolytic	0	2.75	0	2.75	0	2.75	0	2.75
TCGA-D5-6529-01	Cluster 2	Glycolytic	0	1.68	0	1.68	NA	NA	1	1.06
TCGA-F4-6805-01	Cluster 2	Glycolytic	0	2.87	0	2.87	0	2.87	0	2.87
TCGA-AA-3877-01	Cluster 2	Glycolytic	0	2.58	0	2.58	0	2.58	0	2.58
TCGA-AZ-6598-01	Cluster 2	Glycolytic	1	4.12	0	4.12	0	4.12	0	4.12
TCGA-AA-A01Q-01	Cluster 2	Glycolytic	0	0.08	0	0.08	NA	NA	0	0.08
TCGA-D5-6540-01	Cluster 2	Glycolytic	0	1.35	0	1.35	NA	NA	0	1.35
TCGA-AA-3516-01	Cluster 2	Glycolytic	1	1.08	1	1.08	NA	NA	1	1.08
TCGA-D5-5538-01	Cluster 2	Glycolytic	1	4.55	1	4.55	NA	NA	1	2.76
TCGA-AA-3844-01	Cluster 2	Glycolytic	0	1.24	0	1.24	NA	NA	1	1.00
TCGA-G4-6626-01	Cluster 2	Glycolytic	1	3.90	0	3.90	NA	NA	0	3.90
TCGA-D5-6535-01	Cluster 2	Glycolytic	0	1.26	0	1.26	NA	NA	0	1.26
TCGA-AZ-4614-01	Cluster 2	Glycolytic	1	0.47	1	0.47	NA	NA	1	0.47
TCGA-AA-A01V-01	Cluster 2	Glycolytic	0	0.08	0	0.08	NA	NA	0	0.08
TCGA-CA-6718-01	Cluster 2	Glycolytic	1	0.84	1	0.84	1	0.57	1	0.57
TCGA-AA-3538-01	Cluster 2	Glycolytic	0	2.17	0	2.17	0	2.17	0	2.17
TCGA-D5-6930-01	Cluster 2	Glycolytic	0	1.11	0	1.11	NA	NA	0	1.11
TCGA-AA-3930-01	Cluster 2	Glycolytic	1	0.17	1	0.17	NA	NA	1	0.17
TCGA-CK-4952-01	Cluster 2	Glycolytic	0	1.30	0	1.30	NA	NA	0	1.30
TCGA-DM-A1D6-01	Cluster 2	Glycolytic	1	4.16	NA	4.16	NA	NA	0	4.16
TCGA-AA-3673-01	Cluster 2	Glycolytic	0	4.17	0	4.17	0	4.17	0	4.17
TCGA-AA-A00N-01	Cluster 2	Glycolytic	1	0.33	1	0.33	NA	NA	1	0.33
TCGA-AA-3510-01	Cluster 2	Glycolytic	0	5.33	0	5.33	0	5.33	0	5.33
TCGA-A6-4105-01	Cluster 2	Glycolytic	1	1.21	0	1.21	NA	NA	1	1.00
TCGA-CM-5861-01	Cluster 2	Glycolytic	0	1.25	0	1.25	NA	NA	1	0.92

TCGA-CM-6168-01	Cluster 2	Glycolytic	0	1.08	0	1.08	NA	NA	0	1.08
TCGA-AA-A01K-01	Cluster 2	Glycolytic	0	2.58	0	2.58	0	2.58	0	2.58
TCGA-AD-6965-01	Cluster 2	Glycolytic	0	2.21	0	2.21	1	1.78	1	1.78
TCGA-A6-2671-01	Cluster 2	Glycolytic	1	3.65	1	3.65	NA	NA	1	1.47
TCGA-AA-3861-01	Cluster 2	Glycolytic	0	2.50	0	2.50	0	2.50	0	2.50
TCGA-AA-3821-01	Cluster 2	Glycolytic	0	0.08	0	0.08	NA	NA	0	0.08
TCGA-AA-A01X-01	Cluster 2	Glycolytic	0	2.17	0	2.17	NA	NA	1	2.17
TCGA-A6-6138-01	Cluster 2	Glycolytic	0	1.88	0	1.88	NA	NA	0	1.88
TCGA-AA-3860-01	Cluster 2	Glycolytic	0	2.59	0	2.59	0	2.59	0	2.59
TCGA-AA-3525-01	Cluster 2	Glycolytic	0	0.67	0	0.67	NA	NA	0	0.67
TCGA-AA-3697-01	Cluster 2	Glycolytic	0	7.09	0	7.09	NA	NA	0	7.09
TCGA-AA-3950-01	Cluster 2	Glycolytic	0	2.00	0	2.00	0	2.00	0	2.00
TCGA-AD-6895-01	Cluster 2	Glycolytic	0	2.09	0	2.09	0	2.09	0	2.09
TCGA-AA-A02K-01	Cluster 2	Glycolytic	1	1.17	1	1.17	NA	NA	1	0.84
TCGA-G4-6628-01	Cluster 2	Glycolytic	0	6.64	0	6.64	0	6.64	0	6.64
TCGA-AA-3949-01	Cluster 2	Glycolytic	0	2.17	0	2.17	0	2.17	0	2.17
TCGA-AA-3815-01	Cluster 2	Glycolytic	0	2.75	0	2.75	0	2.75	0	2.75
TCGA-A6-6648-01	Cluster 2	Glycolytic	0	2.10	0	2.10	NA	NA	1	1.88
TCGA-CM-6172-01	Cluster 2	Glycolytic	0	0.92	0	0.92	NA	NA	0	0.92
TCGA-F4-6808-01	Cluster 2	Glycolytic	0	2.81	0	2.81	0	2.81	0	2.81
TCGA-AA-3664-01	Cluster 2	Glycolytic	0	4.50	0	4.50	NA	NA	0	4.50
TCGA-AA-3534-01	Cluster 2	Glycolytic	0	2.42	0	2.42	0	2.42	0	2.42
TCGA-DM-A288-01	Cluster 2	Glycolytic	1	1.17	NA	1.17	NA	NA	1	0.88
TCGA-A6-3809-01	Cluster 2	Glycolytic	0	2.73	0	2.73	NA	NA	0	2.73
TCGA-D5-6923-01	Cluster 2	Glycolytic	0	1.04	0	1.04	NA	NA	0	1.04
TCGA-AA-3543-01	Cluster 2	Glycolytic	0	0.08	0	0.08	NA	NA	0	0.08

TCGA-AM-5820-01	Cluster 2	Glycolytic	0	0.04	0	0.04	NA	NA	0	0.04
TCGA-AA-A01R-01	Cluster 2	Glycolytic	0	2.92	0	2.92	NA	NA	1	1.67
TCGA-AY-A69D-01	Cluster 3	Mixed	0	1.49	0	1.49	0	1.49	0	1.49
TCGA-A6-2672-01	Cluster 3	Mixed	0	3.89	0	3.89	0	3.89	0	3.89
TCGA-CA-6715-01	Cluster 3	Mixed	0	1.05	0	1.05	0	1.05	0	1.05
TCGA-D5-7000-01	Cluster 3	Mixed	0	0.85	0	0.85	NA	NA	0	0.85
TCGA-AA-A01C-01	Cluster 3	Mixed	0	1.25	0	1.25	NA	NA	0	1.25
TCGA-AA-3869-01	Cluster 3	Mixed	1	2.25	1	2.25	NA	NA	1	2.25
TCGA-CA-6717-01	Cluster 3	Mixed	0	1.06	0	1.06	0	1.06	0	1.06
TCGA-A6-5661-01	Cluster 3	Mixed	0	2.79	0	2.79	0	2.79	0	2.79
TCGA-AA-3509-01	Cluster 3	Mixed	0	5.25	0	5.25	0	5.25	0	5.25
TCGA-AA-3713-01	Cluster 3	Mixed	0	1.59	0	1.59	NA	NA	0	1.59
TCGA-A6-2677-01	Cluster 3	Mixed	1	2.03	0	2.03	NA	NA	0	2.03
TCGA-QG-A5YV-01	Cluster 3	Mixed	0	3.56	0	3.56	0	3.56	0	3.56
TCGA-AA-3519-01	Cluster 3	Mixed	0	0.76	0	0.76	0	0.76	0	0.76
TCGA-DM-A0X9-01	Cluster 3	Mixed	0	9.98	0	9.98	NA	NA	0	9.98
TCGA-CK-5914-01	Cluster 3	Mixed	0	1.83	0	1.83	NA	NA	0	1.83
TCGA-AA-3819-01	Cluster 3	Mixed	0	2.08	0	2.08	0	2.08	0	2.08
TCGA-CM-4747-01	Cluster 3	Mixed	0	2.08	0	2.08	NA	NA	0	2.08
TCGA-AD-6889-01	Cluster 3	Mixed	1	6.94	0	6.94	1	4.15	1	4.15
TCGA-AA-3531-01	Cluster 3	Mixed	0	2.84	0	2.84	0	2.84	0	2.84
TCGA-AD-6548-01	Cluster 3	Mixed	0	1.78	0	1.78	0	1.78	0	1.78
TCGA-CM-6674-01	Cluster 3	Mixed	0	1.08	0	1.08	NA	NA	0	1.08
TCGA-5M-AAT4-01	Cluster 3	Mixed	1	0.13	1	0.13	NA	NA	1	0.13
TCGA-AA-3989-01	Cluster 3	Mixed	1	0.66	1	0.66	NA	NA	1	0.66
TCGA-CK-6747-01	Cluster 3	Mixed	0	6.91	0	6.91	0	6.91	0	6.91

TCGA-AA-3955-01	Cluster 3	Mixed	0	1.75	0	1.75	0	1.75	0	1.75
TCGA-T9-A92H-01	Cluster 3	Mixed	0	0.99	0	0.99	1	0.22	1	0.22
TCGA-AA-3492-01	Cluster 3	Mixed	1	0.25	0	0.25	NA	NA	0	0.25
TCGA-AA-3848-01	Cluster 3	Mixed	1	0.84	1	0.84	NA	NA	1	0.84
TCGA-CM-4744-01	Cluster 3	Mixed	0	1.67	0	1.67	0	1.67	0	1.67
TCGA-QG-A5YW-01	Cluster 3	Mixed	0	2.45	0	2.45	0	2.45	0	2.45
TCGA-CA-5797-01	Cluster 3	Mixed	0	1.05	0	1.05	0	1.05	0	1.05
TCGA-AA-A01F-01	Cluster 3	Mixed	0	2.67	0	2.67	NA	NA	0	2.67
TCGA-D5-6531-01	Cluster 3	Mixed	0	1.48	0	1.48	NA	NA	0	1.48
TCGA-DM-A28H-01	Cluster 3	Mixed	0	9.76	0	9.76	NA	NA	1	1.33
TCGA-AA-A02Y-01	Cluster 3	Mixed	0	3.33	0	3.33	0	3.33	0	3.33
TCGA-AD-6890-01	Cluster 3	Mixed	0	2.04	0	2.04	0	2.04	0	2.04
TCGA-AA-3982-01	Cluster 3	Mixed	0	2.25	0	2.25	NA	NA	0	2.25
TCGA-AZ-6600-01	Cluster 3	Mixed	1	1.01	1	1.01	NA	NA	1	1.01
TCGA-AA-A00K-01	Cluster 3	Mixed	0	1.50	0	1.50	NA	NA	0	1.50
TCGA-AA-3555-01	Cluster 3	Mixed	0	2.50	0	2.50	1	0.25	1	0.25
TCGA-AA-A01I-01	Cluster 3	Mixed	0	2.58	0	2.58	0	2.58	0	2.58
TCGA-AA-A01S-01	Cluster 3	Mixed	0	0.08	0	0.08	NA	NA	0	0.08
TCGA-A6-5659-01	Cluster 3	Mixed	0	2.54	0	2.54	0	2.54	0	2.54
TCGA-G4-6295-01	Cluster 3	Mixed	0	0.70	0	0.70	NA	NA	0	0.70
TCGA-CM-4750-01	Cluster 3	Mixed	0	0.67	0	0.67	0	0.67	0	0.67
TCGA-AY-6386-01	Cluster 3	Mixed	0	1.48	0	1.48	0	1.48	0	1.48
TCGA-CK-4951-01	Cluster 3	Mixed	1	5.85	0	5.85	NA	NA	1	1.32
TCGA-D5-6927-01	Cluster 3	Mixed	0	0.79	0	0.79	NA	NA	0	0.79
TCGA-CK-6751-01	Cluster 3	Mixed	0	10.36	0	10.36	NA	NA	0	10.36
TCGA-DM-A280-01	Cluster 3	Mixed	1	0.65	NA	0.65	NA	NA	0	0.65

TCGA-AA-3554-01	Cluster 3	Mixed	0	1.50	0	1.50	0	1.50	0	1.50
TCGA-AA-3553-01	Cluster 3	Mixed	0	2.00	0	2.00	0	2.00	0	2.00
TCGA-AA-3663-01	Cluster 3	Mixed	0	0.58	0	0.58	0	0.58	0	0.58
TCGA-DM-A0XD-01	Cluster 3	Mixed	1	2.04	NA	2.04	NA	NA	1	1.33
TCGA-AA-A01G-01	Cluster 3	Mixed	0	1.00	0	1.00	0	1.00	0	1.00
TCGA-AA-3855-01	Cluster 3	Mixed	0	2.67	0	2.67	NA	NA	0	2.67
TCGA-G4-6311-01	Cluster 3	Mixed	0	3.28	0	3.28	NA	NA	0	3.28
TCGA-DM-A1HB-01	Cluster 3	Mixed	0	11.30	0	11.30	NA	NA	0	11.30
TCGA-AA-A00U-01	Cluster 3	Mixed	0	1.42	0	1.42	0	1.42	0	1.42
TCGA-AA-A00A-01	Cluster 3	Mixed	0	3.17	0	3.17	NA	NA	0	3.17
TCGA-F4-6856-01	Cluster 3	Mixed	0	2.94	0	2.94	0	2.94	0	2.94
TCGA-G4-6588-01	Cluster 3	Mixed	0	2.18	0	2.18	NA	NA	0	2.18
TCGA-A6-5667-01	Cluster 3	Mixed	0	2.43	0	2.43	0	2.43	0	2.43
TCGA-CM-6677-01	Cluster 3	Mixed	0	0.92	0	0.92	NA	NA	0	0.92
TCGA-AZ-4615-01	Cluster 3	Mixed	0	2.75	0	2.75	NA	NA	1	1.88
TCGA-D5-6931-01	Cluster 3	Mixed	0	1.00	0	1.00	NA	NA	0	1.00
TCGA-AZ-4313-01	Cluster 3	Mixed	0	6.33	0	6.33	0	6.33	0	6.33
TCGA-CM-6675-01	Cluster 3	Mixed	0	1.09	0	1.09	NA	NA	1	0.92
TCGA-NH-A6GB-01	Cluster 3	Mixed	0	1.30	0	1.30	NA	NA	0	1.30
TCGA-A6-6653-01	Cluster 3	Mixed	0	2.03	0	2.03	0	2.03	0	2.03
TCGA-CM-4746-01	Cluster 3	Mixed	0	3.08	0	3.08	0	3.08	0	3.08
TCGA-QG-A5Z2-01	Cluster 3	Mixed	0	2.61	0	2.61	0	2.61	0	2.61
TCGA-AZ-6601-01	Cluster 3	Mixed	1	8.33	1	8.33	NA	NA	1	6.22
TCGA-AA-3811-01	Cluster 3	Mixed	1	0.84	1	0.84	NA	NA	1	0.84
TCGA-AA-A00E-01	Cluster 3	Mixed	0	2.50	0	2.50	0	2.50	0	2.50
TCGA-DM-A28K-01	Cluster 3	Mixed	0	8.19	0	8.19	NA	NA	0	8.19

TCGA-AA-3532-01	Cluster 3	Mixed	0	2.42	0	2.42	NA	NA	0	2.42
TCGA-DM-A28C-01	Cluster 3	Mixed	1	6.78	NA	6.78	NA	NA	1	5.28
TCGA-AA-3666-01	Cluster 3	Mixed	1	0.17	1	0.17	NA	NA	1	0.17
TCGA-AA-3561-01	Cluster 3	Mixed	0	1.16	0	1.16	0	1.16	0	1.16
TCGA-D5-6898-01	Cluster 3	Mixed	0	0.63	0	0.63	NA	NA	0	0.63
TCGA-AA-3526-01	Cluster 3	Mixed	0	1.59	0	1.59	0	1.59	0	1.59
TCGA-AA-3831-01	Cluster 3	Mixed	0	1.50	0	1.50	0	1.50	0	1.50
TCGA-A6-6650-01	Cluster 3	Mixed	0	1.72	0	1.72	NA	NA	0	1.72
TCGA-A6-2676-01	Cluster 3	Mixed	1	3.58	0	3.58	0	3.58	0	3.58
TCGA-G4-6322-01	Cluster 3	Mixed	0	2.17	0	2.17	NA	NA	1	2.16
TCGA-AA-3524-01	Cluster 3	Mixed	0	3.00	0	3.00	0	3.00	0	3.00
TCGA-AY-A54L-01	Cluster 3	Mixed	0	1.44	0	1.44	1	1.08	1	1.08
TCGA-NH-A50T-01	Cluster 3	Mixed	0	1.52	0	1.52	0	1.52	0	1.52
TCGA-QL-A97D-01	Cluster 3	Mixed	0	1.82	0	1.82	0	1.82	0	1.82
TCGA-AA-3675-01	Cluster 3	Mixed	0	3.92	0	3.92	0	3.92	0	3.92
TCGA-AA-3688-01	Cluster 3	Mixed	0	1.58	0	1.58	NA	NA	0	1.58
TCGA-D5-6533-01	Cluster 3	Mixed	0	2.12	0	2.12	0	2.12	0	2.12
TCGA-AZ-4684-01	Cluster 3	Mixed	0	5.42	0	5.42	NA	NA	1	1.13
TCGA-4T-AA8H-01	Cluster 3	Mixed	0	1.05	0	1.05	0	1.05	0	1.05
TCGA-AA-A010-01	Cluster 3	Mixed	0	2.92	0	2.92	0	2.92	0	2.92
TCGA-A6-2678-01	Cluster 3	Mixed	0	3.52	0	3.52	0	3.52	0	3.52
TCGA-AA-3678-01	Cluster 3	Mixed	0	3.92	0	3.92	0	3.92	0	3.92
TCGA-AZ-6608-01	Cluster 3	Mixed	1	0.16	NA	0.16	NA	NA	0	0.16
TCGA-AA-3841-01	Cluster 3	Mixed	0	3.08	0	3.08	0	3.08	0	3.08
TCGA-AY-5543-01	Cluster 3	Mixed	0	2.75	0	2.75	NA	NA	0	2.75
TCGA-AA-3520-01	Cluster 3	Mixed	0	2.00	0	2.00	NA	NA	0	2.00

TCGA-AA-A01Z-01	Cluster 3	Mixed	0	3.08	0	3.08	NA	NA	1	3.08
TCGA-A6-2681-01	Cluster 3	Mixed	0	3.80	0	3.80	1	3.15	1	3.15
TCGA-AA-A00Z-01	Cluster 3	Mixed	0	1.83	0	1.83	0	1.83	0	1.83
TCGA-RU-A8FL-01	Cluster 3	Mixed	0	3.22	0	3.22	NA	NA	1	0.70
TCGA-AA-3856-01	Cluster 3	Mixed	0	0.08	0	0.08	NA	NA	0	0.08
TCGA-G4-6315-01	Cluster 3	Mixed	0	5.16	0	5.16	NA	NA	0	5.16
TCGA-AA-3866-01	Cluster 3	Mixed	0	1.42	0	1.42	0	1.42	0	1.42
TCGA-AA-3556-01	Cluster 3	Mixed	0	1.92	0	1.92	NA	NA	0	1.92
TCGA-CK-5913-01	Cluster 3	Mixed	0	4.28	0	4.28	0	4.28	0	4.28
TCGA-AA-3979-01	Cluster 3	Mixed	0	2.00	0	2.00	0	2.00	0	2.00
TCGA-AD-6963-01	Cluster 3	Mixed	0	2.28	0	2.28	0	2.28	0	2.28
TCGA-DM-A1D4-01	Cluster 3	Mixed	1	7.73	NA	7.73	NA	NA	1	5.26
TCGA-D5-6920-01	Cluster 3	Mixed	0	1.03	0	1.03	NA	NA	0	1.03
TCGA-A6-5666-01	Cluster 3	Mixed	0	2.73	0	2.73	1	1.39	1	1.39
TCGA-A6-2684-01	Cluster 3	Mixed	0	3.09	0	3.09	NA	NA	1	2.67
TCGA-CA-5255-01	Cluster 3	Mixed	0	1.03	0	1.03	0	1.03	0	1.03
TCGA-AA-A00W-01	Cluster 3	Mixed	0	1.25	0	1.25	NA	NA	0	1.25
TCGA-A6-6140-01	Cluster 3	Mixed	0	2.01	0	2.01	0	2.01	0	2.01
TCGA-AY-6197-01	Cluster 3	Mixed	0	1.79	0	1.79	0	1.79	0	1.79
TCGA-AD-5900-01	Cluster 3	Mixed	0	1.01	0	1.01	0	1.01	0	1.01
TCGA-A6-2682-01	Cluster 3	Mixed	1	1.16	1	1.16	NA	NA	1	1.04
TCGA-DM-A28A-01	Cluster 3	Mixed	1	2.21	NA	2.21	NA	NA	0	2.21
TCGA-AA-3495-01	Cluster 3	Mixed	0	3.09	0	3.09	0	3.09	0	3.09
TCGA-DM-A0XF-01	Cluster 3	Mixed	1	3.18	NA	3.18	NA	NA	0	3.18
TCGA-G4-6309-01	Cluster 3	Mixed	0	7.12	0	7.12	1	5.96	1	5.96
TCGA-AA-3842-01	Cluster 3	Mixed	0	3.08	0	3.08	1	1.08	1	1.08

TCGA-AA-3655-01	Cluster 3	Mixed	0	5.08	0	5.08	0	5.08	0	5.08
TCGA-D5-6539-01	Cluster 3	Mixed	0	1.04	0	1.04	NA	NA	0	1.04
TCGA-AA-3548-01	Cluster 3	Mixed	0	2.83	0	2.83	0	2.83	0	2.83
TCGA-AD-6888-01	Cluster 3	Mixed	1	1.29	1	1.29	1	0.94	1	0.94
TCGA-CK-4950-01	Cluster 3	Mixed	0	7.12	0	7.12	0	7.12	0	7.12
TCGA-AA-3977-01	Cluster 3	Mixed	0	2.08	0	2.08	0	2.08	0	2.08
TCGA-AA-A02J-01	Cluster 3	Mixed	1	0.42	1	0.42	NA	NA	1	0.42
TCGA-AA-3681-01	Cluster 3	Mixed	0	0.50	0	0.50	0	0.50	0	0.50
TCGA-AZ-4315-01	Cluster 3	Mixed	0	4.87	0	4.87	NA	NA	0	4.87
TCGA-AA-3560-01	Cluster 3	Mixed	0	1.67	0	1.67	0	1.67	0	1.67
TCGA-AA-3858-01	Cluster 3	Mixed	0	2.59	0	2.59	0	2.59	0	2.59
TCGA-AA-3864-01	Cluster 3	Mixed	0	4.42	0	4.42	0	4.42	0	4.42
TCGA-AZ-6606-01	Cluster 3	Mixed	1	0.98	1	0.98	NA	NA	1	0.98
TCGA-D5-6532-01	Cluster 3	Mixed	0	1.52	0	1.52	NA	NA	0	1.52
TCGA-AA-A029-01	Cluster 3	Mixed	0	4.33	0	4.33	0	4.33	0	4.33
TCGA-D5-5540-01	Cluster 3	Mixed	0	4.67	0	4.67	0	4.67	0	4.67
TCGA-A6-6780-01	Cluster 3	Mixed	0	1.68	0	1.68	0	1.68	0	1.68
TCGA-DM-A28F-01	Cluster 3	Mixed	1	3.00	NA	3.00	NA	NA	0	3.00
TCGA-AA-3488-01	Cluster 3	Mixed	1	0.42	1	0.42	NA	NA	1	0.42
TCGA-DM-A1DA-01	Cluster 3	Mixed	1	0.62	NA	0.62	NA	NA	0	0.62
TCGA-AA-3980-01	Cluster 3	Mixed	0	2.25	0	2.25	0	2.25	0	2.25
TCGA-A6-2680-01	Cluster 3	Mixed	0	2.93	0	2.93	0	2.93	0	2.93
TCGA-AA-3562-01	Cluster 4	Cholesterogenic	0	1.67	0	1.67	NA	NA	0	1.67
TCGA-A6-5660-01	Cluster 4	Cholesterogenic	0	2.43	0	2.43	0	2.43	0	2.43
TCGA-G4-6321-01	Cluster 4	Cholesterogenic	0	1.84	0	1.84	NA	NA	0	1.84
TCGA-AA-3956-01	Cluster 4	Cholesterogenic	0	2.84	0	2.84	NA	NA	0	2.84

TCGA-D5-5541-01	Cluster 4	Cholesterogenic	0	4.66	0	4.66	0	4.66	0	4.66
TCGA-A6-2683-01	Cluster 4	Cholesterogenic	1	1.38	1	1.38	NA	NA	1	0.82
TCGA-G4-6306-01	Cluster 4	Cholesterogenic	0	3.72	0	3.72	NA	NA	0	3.72
TCGA-AA-3939-01	Cluster 4	Cholesterogenic	0	1.08	0	1.08	NA	NA	0	1.08
TCGA-A6-4107-01	Cluster 4	Cholesterogenic	0	2.70	0	2.70	0	2.70	0	2.70
TCGA-QG-A5Z1-01	Cluster 4	Cholesterogenic	1	0.70	0	0.70	NA	NA	0	0.70
TCGA-AA-3544-01	Cluster 4	Cholesterogenic	0	1.17	0	1.17	0	1.17	0	1.17
TCGA-CA-5256-01	Cluster 4	Cholesterogenic	0	1.04	0	1.04	0	1.04	0	1.04
TCGA-AA-3846-01	Cluster 4	Cholesterogenic	0	1.42	0	1.42	NA	NA	0	1.42
TCGA-AA-3976-01	Cluster 4	Cholesterogenic	0	2.17	0	2.17	0	2.17	0	2.17
TCGA-DM-A282-01	Cluster 4	Cholesterogenic	0	11.60	0	11.60	NA	NA	0	11.60
TCGA-A6-6137-01	Cluster 4	Cholesterogenic	0	2.26	0	2.26	0	2.26	0	2.26
TCGA-G4-6303-01	Cluster 4	Cholesterogenic	1	5.49	1	5.49	NA	NA	1	2.39
TCGA-CM-6676-01	Cluster 4	Cholesterogenic	0	0.92	0	0.92	NA	NA	0	0.92
TCGA-AA-3986-01	Cluster 4	Cholesterogenic	0	1.59	0	1.59	0	1.59	0	1.59
TCGA-AA-3660-01	Cluster 4	Cholesterogenic	0	6.51	0	6.51	0	6.51	0	6.51
TCGA-G4-6314-01	Cluster 4	Cholesterogenic	0	2.99	0	2.99	NA	NA	1	0.10
TCGA-AA-3685-01	Cluster 4	Cholesterogenic	0	3.09	0	3.09	0	3.09	0	3.09
TCGA-AA-3514-01	Cluster 4	Cholesterogenic	0	0.08	0	0.08	NA	NA	0	0.08
TCGA-DM-A1D7-01	Cluster 4	Cholesterogenic	1	1.11	NA	1.11	NA	NA	1	0.22
TCGA-AA-3667-01	Cluster 4	Cholesterogenic	0	1.17	0	1.17	0	1.17	0	1.17
TCGA-4N-A93T-01	Cluster 4	Cholesterogenic	0	0.40	0	0.40	NA	NA	0	0.40
TCGA-D5-5537-01	Cluster 4	Cholesterogenic	1	3.78	1	3.78	1	1.98	1	1.98
TCGA-CM-6678-01	Cluster 4	Cholesterogenic	0	0.92	0	0.92	NA	NA	1	0.00
TCGA-QG-A5YX-01	Cluster 4	Cholesterogenic	0	2.75	0	2.75	0	2.75	0	2.75
TCGA-DM-A28G-01	Cluster 4	Cholesterogenic	1	5.07	NA	5.07	NA	NA	0	5.07

TCGA-CM-5868-01	Cluster 4	Cholesterogenic	0	1.42	0	1.42	NA	NA	0	1.42
TCGA-A6-A5ZU-01	Cluster 4	Cholesterogenic	0	0.80	0	0.80	0	0.80	0	0.80
TCGA-AY-A8YK-01	Cluster 4	Cholesterogenic	0	1.57	0	1.57	NA	NA	0	1.57
TCGA-A6-3807-01	Cluster 4	Cholesterogenic	0	2.89	0	2.89	NA	NA	0	2.89
TCGA-NH-A8F8-01	Cluster 4	Cholesterogenic	1	1.40	1	1.40	NA	NA	1	1.40
TCGA-DM-A285-01	Cluster 4	Cholesterogenic	1	0.49	NA	0.49	NA	NA	0	0.49
TCGA-AZ-4308-01	Cluster 4	Cholesterogenic	0	9.11	0	9.11	0	9.11	0	9.11
TCGA-CM-5860-01	Cluster 4	Cholesterogenic	0	2.67	0	2.67	NA	NA	0	2.67
TCGA-CM-6163-01	Cluster 4	Cholesterogenic	0	1.17	0	1.17	NA	NA	0	1.17
TCGA-AY-4070-01	Cluster 4	Cholesterogenic	1	1.36	1	1.36	NA	NA	1	0.51
TCGA-CM-4748-01	Cluster 4	Cholesterogenic	0	2.17	0	2.17	0	2.17	0	2.17
TCGA-CM-4751-01	Cluster 4	Cholesterogenic	0	2.25	0	2.25	0	2.25	0	2.25
TCGA-AZ-6603-01	Cluster 4	Cholesterogenic	1	2.46	0	2.46	0	2.46	0	2.46
TCGA-AA-3851-01	Cluster 4	Cholesterogenic	0	2.76	0	2.76	NA	NA	0	2.76
TCGA-AA-3679-01	Cluster 4	Cholesterogenic	0	1.25	0	1.25	NA	NA	0	1.25
TCGA-AA-3994-01	Cluster 4	Cholesterogenic	0	2.25	0	2.25	0	2.25	0	2.25
TCGA-G4-6317-01	Cluster 4	Cholesterogenic	0	3.00	0	3.00	NA	NA	1	1.62
TCGA-AA-3941-01	Cluster 4	Cholesterogenic	0	2.00	0	2.00	NA	NA	0	2.00
TCGA-SS-A7HO-01	Cluster 4	Cholesterogenic	0	5.01	0	5.01	1	1.39	1	1.39
TCGA-CM-5341-01	Cluster 4	Cholesterogenic	0	2.42	0	2.42	0	2.42	0	2.42
TCGA-AA-A024-01	Cluster 4	Cholesterogenic	1	3.25	0	3.25	0	3.25	0	3.25
TCGA-D5-6929-01	Cluster 4	Cholesterogenic	0	1.12	0	1.12	NA	NA	0	1.12
TCGA-A6-6782-01	Cluster 4	Cholesterogenic	0	1.69	0	1.69	0	1.69	0	1.69
TCGA-CA-6716-01	Cluster 4	Cholesterogenic	0	1.02	0	1.02	0	1.02	0	1.02
TCGA-AA-3542-01	Cluster 4	Cholesterogenic	0	1.08	0	1.08	0	1.08	0	1.08
TCGA-CM-6166-01	Cluster 4	Cholesterogenic	0	1.83	0	1.83	NA	NA	0	1.83

TCGA-AA-A01T-01	Cluster 4	Cholesterogenic	0	2.75	0	2.75	0	2.75	0	2.75
TCGA-A6-6141-01	Cluster 4	Cholesterogenic	0	0.70	0	0.70	NA	NA	0	0.70
TCGA-AA-A02E-01	Cluster 4	Cholesterogenic	1	0.25	1	0.25	NA	NA	1	0.25
TCGA-D5-6932-01	Cluster 4	Cholesterogenic	0	0.95	0	0.95	NA	NA	0	0.95
TCGA-CK-5912-01	Cluster 4	Cholesterogenic	1	4.09	0	4.09	0	4.09	0	4.09
TCGA-A6-5662-01	Cluster 4	Cholesterogenic	0	1.97	0	1.97	NA	NA	1	1.36
TCGA-A6-2679-01	Cluster 4	Cholesterogenic	0	3.74	0	3.74	0	3.74	0	3.74
TCGA-DM-A1D0-01	Cluster 4	Cholesterogenic	0	10.89	0	10.89	NA	NA	0	10.89
TCGA-G4-6307-01	Cluster 4	Cholesterogenic	0	4.59	0	4.59	NA	NA	0	4.59
TCGA-AA-3867-01	Cluster 4	Cholesterogenic	0	2.00	0	2.00	NA	NA	1	1.08
TCGA-CA-5796-01	Cluster 4	Cholesterogenic	0	1.03	0	1.03	0	1.03	0	1.03
TCGA-AA-3970-01	Cluster 4	Cholesterogenic	0	3.00	0	3.00	0	3.00	0	3.00
TCGA-A6-3808-01	Cluster 4	Cholesterogenic	0	2.78	0	2.78	NA	NA	0	2.78
TCGA-F4-6569-01	Cluster 4	Cholesterogenic	0	2.98	0	2.98	0	2.98	0	2.98
TCGA-AA-3837-01	Cluster 4	Cholesterogenic	0	3.25	0	3.25	0	3.25	0	3.25
TCGA-AZ-5403-01	Cluster 4	Cholesterogenic	1	5.23	1	5.23	NA	NA	1	0.83

Table S4. Cancer–immunity cycle activity was evaluated among metabolic subtypes by ssGSEA.

**Step**	***p* value**	**Significance**
Release_of_cancer_cell_antigens	3.67E-04	< 0.005
Cancer_antigen_presentation	0.3640	No significance
Priming_and_activation	0.1140	No significance
T_cell_recruiting	4.50E-04	< 0.005
CD4_T_cell_recruiting	2.51E-05	< 0.001
CD8_T_cell_recruiting	1.10E-05	< 0.001
Th1_cell_recruiting	0.0718	No significance
Dendritic_cell_recruiting	0.0067	< 0.01
Th22_cell_recruiting	2.19E-04	< 0.005
Macrophage_recruiting	0.00106	< 0.01
Monocyte_recruiting	0.6020	No significance
Neutrophil_recruiting	2.73E-04	< 0.005
NK_cell_recruiting	0.0019	< 0.01
Eosinophil_recruiting	0.0547	No significance
Basophil_recruiting	0.3270	No significance
Th17_cell_recruiting	0.0072	< 0.01
B_cell_recruiting	0.07830	No significance
Th2_cell_recruiting	0.6960	No significance
Treg_cell_recruiting	0.3890	No significance
MDSC_recruiting	0.0196	< 0.05
Infiltration_of_immune_cells_into_tumors	2.19E-06	< 0.001
Recognition_of _cancer_cells_by_T_cells	0.1030	No significance
Killing_of_cancer_cells	0.0781	No significance

Table S5. Activities of cancer–immunity cycle were evaluated between glycolytic and cholesterogenic groups by ssGSEA.

**Step**	***p* value**	**Significance**
Release_of_cancer_cell_antigens	3.83E-04	< 0.005
Cancer_antigen_presentation	0.0765	No significance
Priming_and_activation	0.1490	No significance
T_cell_recruiting	3.88E-04	< 0.005
CD4_T_cell_recruiting	0.6290	No significance
CD8_T_cell_recruiting	8.67E-07	< 0.001
Th1_cell_recruiting	0.0106	< 0.05
Dendritic_cell_recruiting	0.0356	< 0.05
Th22_cell_recruiting	0.9590	No significance
Macrophage_recruiting	0.0051	< 0.01
Monocyte_recruiting	0.2350	No significance
Neutrophil_recruiting	5.34E-04	< 0.005
NK_cell_recruiting	2.51E-04	< 0.005
Eosinophil_recruiting	0.1600	No significance
Basophil_recruiting	0.1130	No significance
Th17_cell_recruiting	0.4410	No significance
B_cell_recruiting	0.0808	No significance
Th2_cell_recruiting	0.9130	No significance
Treg_cell_recruiting	0.7390	No significance
MDSC_recruiting	0.0917	No significance
Infiltration_of_immune_cells_into_tumors	0.9360	No significance
Recognition_of _cancer_cells_by_T_cells	0.0390	< 0.05
Killing_of_cancer_cells	0.8170	No significance

Table S6. Co-occurrence of mutations among SYNE1, FAT4, TTN, and TP53.

Gene 1	Gene2	*p* value	Odds Ratio	Event	Pair	Event ratio
SYNE1	FAT4	0.0098	2.8505	Co_Occurence	FAT4, SYNE1	21/45
TTN	FAT4	0.0159	3.1331	Co_Occurence	FAT4, TTN	35/64
SYNE1	TTN	0.0495	2.4134	Co_Occurence	SYNE1, TTN	36/65
TP53	FAT4	0.193	0.603	Mutually_Exclusive	FAT4, TP53	20/78
SYNE1	TP53	0.2702	0.6399	Mutually_Exclusive	SYNE1, TP53	22/77
TP53	TTN	0.7112	1.1802	Co_Occurence	TP53, TTN	53/62

Table S7. Diagnostic efficacy (evaluated AUC value) of metabolism-related genes.

Hub gene	AUC value	95% Confidence Interval
GGH	0.793	0.738 - 0.840
CACNG4	0.642	0.594 - 0.690
MME	0.816	0.741 - 0.883
SLC30A2	0.594	0.538 - 0.691
CKMT2	0.64	0.587 - 0.687
SYN3	0.534	0.467 - 0.597
SLC22A31	0.481	0.416 - 0.553

Table S8. Primer pair sequences using in RT-qPCR.

Species	Gene	Ensembl ID	Sequence (Forward/Reverse)
Human	GGH	ENSG00000137563	5'- GGCATCCAGAGAAAGCACCT - 3'<br>5' - TGCGGTTTTCACAGCATTAGG - 3'
	PKM	ENSG00000067225	5' - CCTGATAGCTCGTGAGGCTG - 3'<br>5' - GTGGAGTGACTTGAGGCTCG - 3'
	GLUT1	ENSG00000117394	5' - CTCCGGTATCGTCAACACG - 3'<br>5' - CCACTTCAAAGAAGGCCACA - 3'
	LDHA	ENSG00000134333	5' - ATGGCAACTCTAAAGGATCAGC - 3'<br>5' - CCAACCCCAACAACTGTAATCT - 3'
	β-actin	ENSG00000111640	5' - CATGTACGTTGCTATCCAGGC - 3'<br>5' - CTCCTTAATGTCACGCACGAT - 3'

Table S9. Relationship between GGH expression and clinical characteristics with overall survivals were evaluated by univariate and multivariate Cox regression analysis.

Characteristics	Univariate Cox			Multivariate Cox		
	**Hazard ratio**	**95% CI**	***p* value**	**Hazard ratio**	**95% CI**	***p* value**
Gender	0.928	0.633 - 1.362	0.704			
Age	1.012	0.994 - 1.029	0.187			
Primary colon cancer site	0.809	0.553 - 1.184	0.276			
T stage	0.686	0.566 - 0.831	<0.001	0.917	0.599 - 1.404	0.690
N stage	0.632	0.498 - 0.802	<0.001	0.788	0.477 - 1.302	0.353
M stage	1.247	0.836 - 1.861	0.279			
KRAS mutation	1.815	1.220 - 2.700	0.003	1.374	0.881 - 2.143	0.162
Expression of GGH2	0.981	0.972 - 0.990	<0.001	0.989	0.979 - 1.000	0.041
